# Syntheses and crystal structures of three tri­phenyl­sulfonium halometallate salts of zinc, cadmium and mercury

**DOI:** 10.1107/S2056989025002245

**Published:** 2025-03-27

**Authors:** Rylan Artis, Elizabeth Heyward, Naomi Reyes, Kaitlyn Van Ostenbridge, Will E. Lynch, Clifford W. Padgett

**Affiliations:** a Department of Biochemistry Chemistry and Physics Georgia Southern University, Armstrong Campus 11935 Abercorn Street Savannah GA 31419 USA; University of Aberdeen, United Kingdom

**Keywords:** crystal structure, tri­phenyl­sulfonium ion, salts

## Abstract

Bis(tri­phenyl­sulfonium) tetra­chlorido­zinc(II), (C_18_H_15_S)_2_[ZnCl_4_] (**I**), bis­(tri­phenyl­sulfonium) tetra­chlorido­cadmium(II), (C_18_H_15_S)_2_[CdCl_4_] (**II**), and bis­(tri­phenyl­sulfonium) tetra­chlorido­mercury(II) methanol monosolvate, (C_18_H_15_S)_2_[HgCl_4_]·CH_3_OH (**III**), each crystallize in the monoclinic space group *P*2_1_/*n*. In all three structures, there are two crystallographically independent tri­phenyl­sulfonium (TPS) cations per asymmetric unit, each adopting a distorted trigonal–pyramidal geometry about the S atom (S—C bond lengths in the 1.77–1.80 Å range and C—S—C angles of 100–107°). The [*M*Cl_4_]^2–^ anions (*M* = Zn^2+^, Cd^2+^, Hg^2+^) are tetra­hedral; their M—Cl bond lengths systematically increase from Zn^2+^ to Hg^2+^, consistent with the larger ionic radius of the heavier metal. Hirshfeld surface analyses show that H⋯H and H⋯C contacts dominate the TPS cation environments, whereas H⋯Cl and S⋯*M* inter­actions anchor each [*M*Cl_4_]^2–^ anion to two surrounding TPS cations. Weak C—H⋯Cl hydrogen bonds, as well as inversion-centered π–π stacking, generate layers in (**I**) and (**II**) and dimeric [(TPS)_2_–HgCl_4_]_2_ assemblies in (**III**).

## Abstract 2

The crystal structures of three salts of the C_18_H_15_S^+^ tri­phenyl­sulfonium ion are reported, namely, bis­(tri­phenyl­sulfonium) tetra­chloro­zinc(II), bis­(tri­phenyl­sulfonium) tetra­chloro­cadmium(II) and bis­(tri­phenyl­sulfonium) tetra­chloro­mercury(II) methanol monosolvate.

## 1. Chemical context

Tri­phenyl­sulfonium (TPS) salts are versatile organosulfur compounds with applications across a variety of chemical and industrial processes. They are widely utilized as photoinitiators in photochemical processes, particularly for the polymerization of ep­oxy resins and other photoresist mat­erials. The basis of their photoinitiating activity lies in their direct or sensitized photolysis, which results in the release of a reactive proton and cleavage of the C—S bond in the tri­phenyl­sulfonium cation. This reaction initiates solubility-changing processes such as cationic polymerization or acid-catalyzed cleavage, enabling desired modifications in material characteristics (Petsalakis *et al.*, 2014 ▸).

Beyond photoinitiation, TPS salts are a subject of inter­est in photochemistry due to their role as photoacid generators, producing acids in response to light exposure (Ohmori *et al.*, 1998 ▸). This unique property makes them valuable for photolithography and the production of semiconductor devices, including computer chips (Kwon *et al.*, 2014 ▸; Wang *et al.*, 2023 ▸). Furthermore, tri­phenyl­sulfonium ions have been studied for their role in inhibiting mitochondrial oxidative phospho­rylation and adenosine triphosphate activity (Barrett *et al.*, 1976 ▸), as well as for their use in exciton emission applications in anti-counterfeiting technologies (Luo *et al.*, 2022*b* ▸).

In this study, we report the crystal structures of three new TPS salts of group-12 complex ions: bis­(tri­phenyl­sulfonium) tetra­chloro­zincate (**I**), bis­(tri­phenyl­sulfonium) tetra­chloro­cadmate (**II**), and bis­(tri­phenyl­sulfonium) tetra­chloro­mercurate methanol monosolvate (**III**). These structures provide valuable insights into packing arrangements and ionic inter­actions, highlighting the influence of metal halide anions on the stability and properties of the tri­phenyl­sulfonium cation.
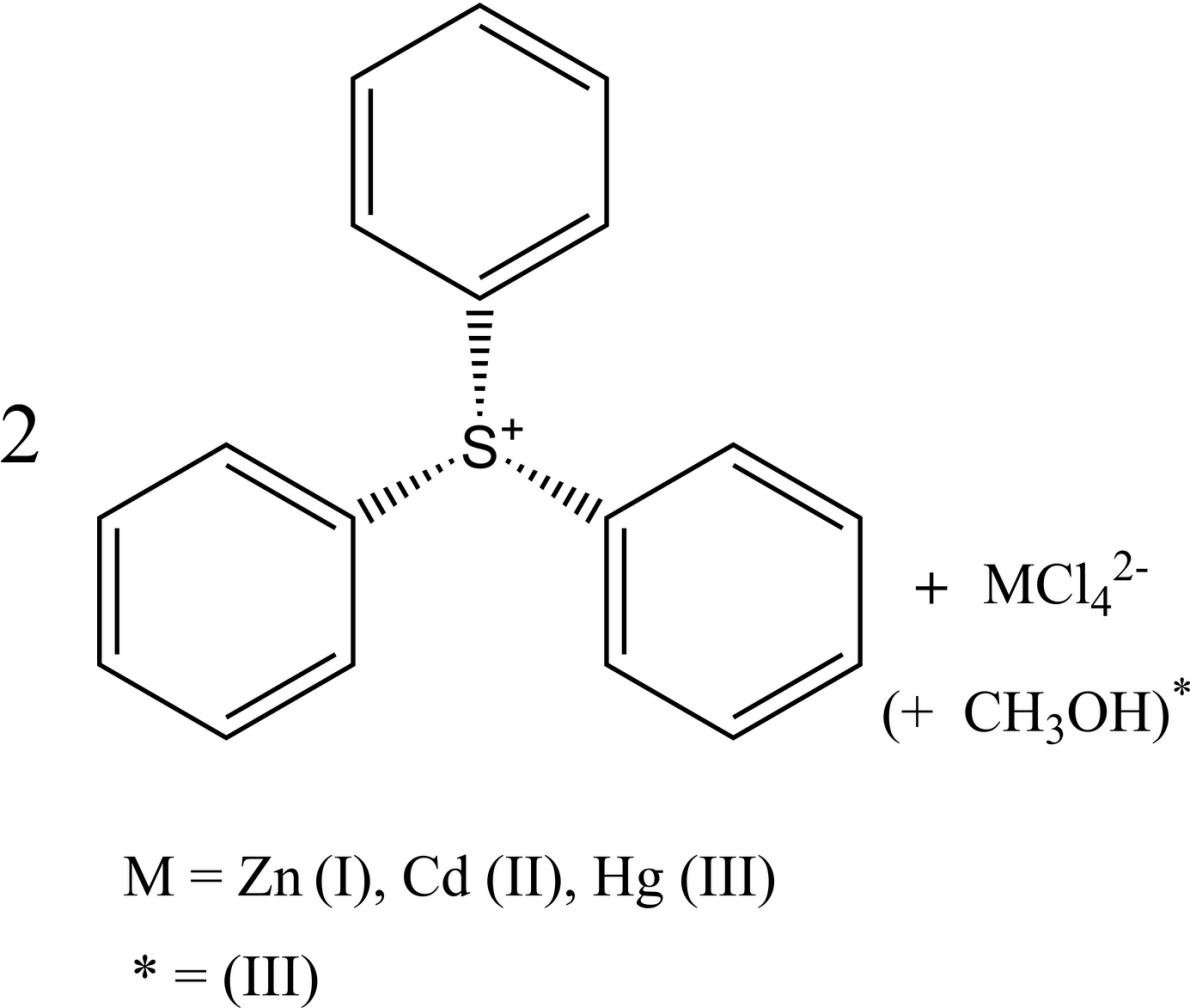


## 2. Structural commentary

**Figure 1:**
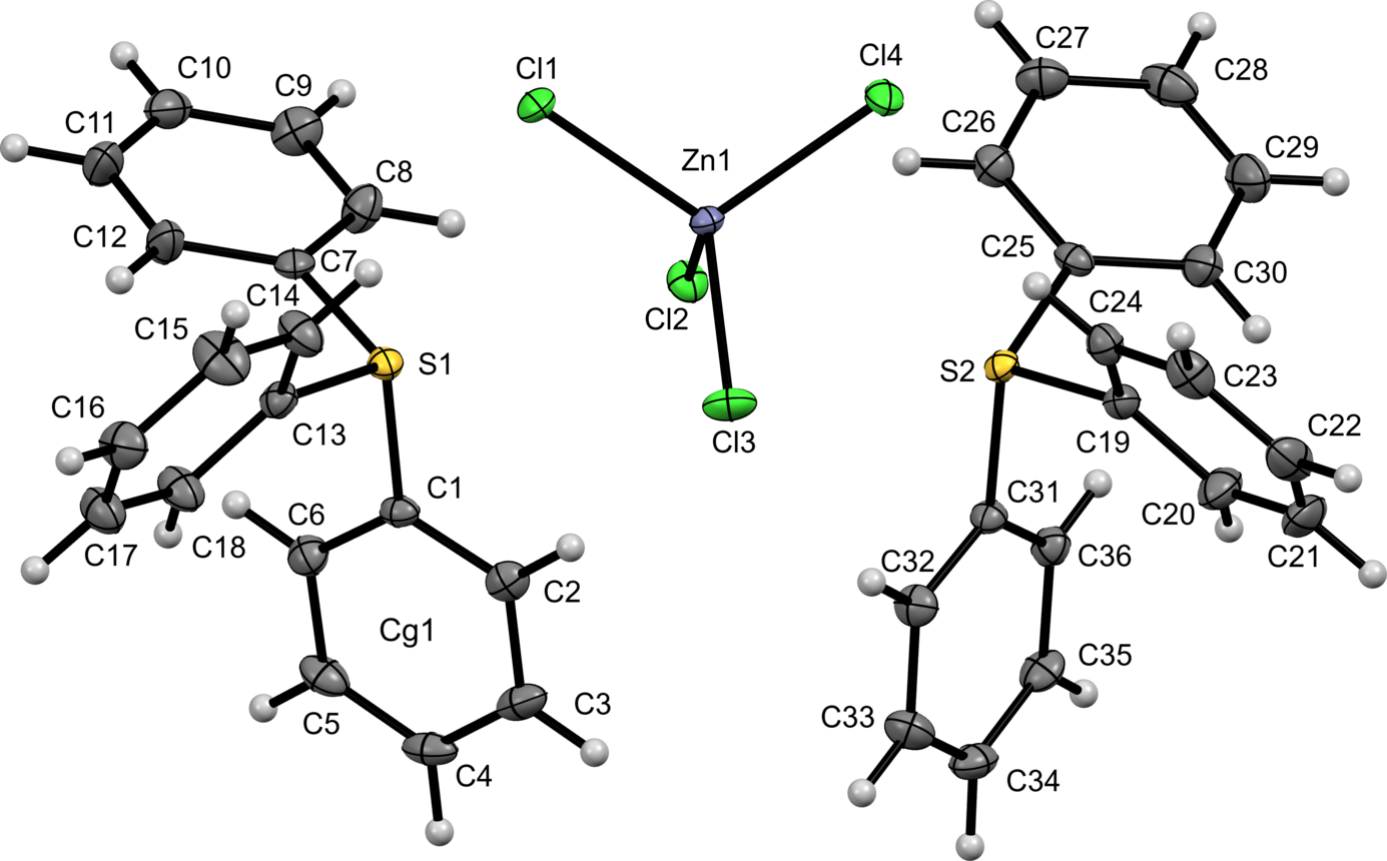
The mol­ecular structure of (**I**) with displacement ellipsoids drawn at the 50% probability level.

Compound (**I**) crystallizes in the monoclinic space group *P*2_1_/*n*. The asymmetric unit of [TPS]_2_[ZnCl_4_] comprises two crystallographically independent C_18_H_15_S^+^ tri­phenyl­sulfonium (TPS^+^) cations and one [ZnCl_4_]^2–^ anion (Fig. 1 ▸). Each sulfonium center exhibits a distorted trigonal–pyramidal geometry. In the first cation (containing S1), the S—C bond lengths lie between 1.7784 (18) and 1.7919 (19) Å, while the C—S—C angles range from 103.45 (9) to 104.88 (9)°. In the second cation (containg S2), the S—C distances span 1.7868 (18)–1.7879 (19) Å, and the C—S—C angles vary from 100.78 (9) to 106.27 (9)°. The [ZnCl_4_]^2−^ anion adopts a slightly distorted tetra­hedral arrangement, with Zn—Cl bond lengths of 2.2615 (5)—2.2863 (5) Å and Cl—Zn—Cl angles in the 105.247 (19)–112.522 (19)° range.

**Figure 2:**
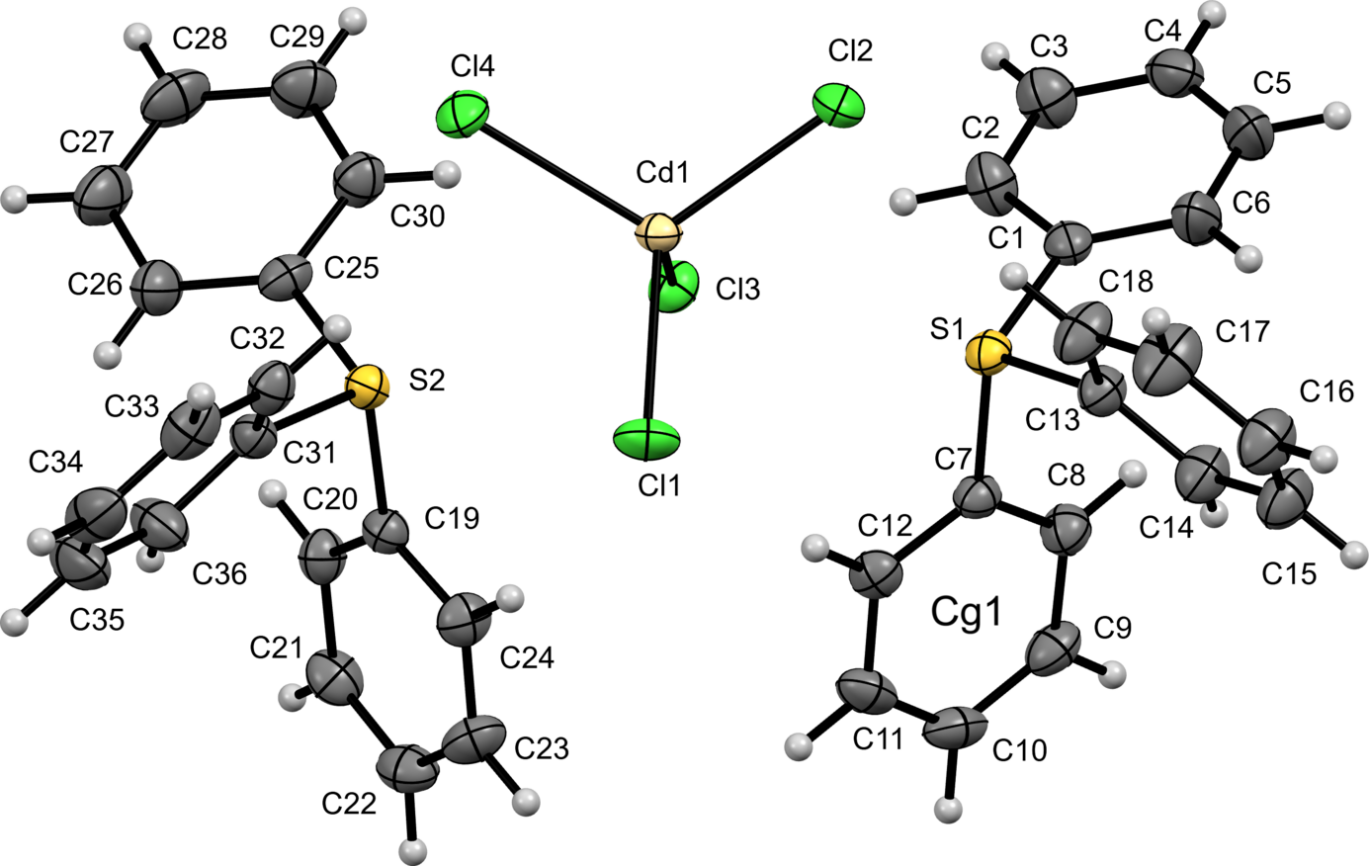
The mol­ecular structure of (**II**) with displacement ellipsoids drawn at the 50% probability level.

Compound (**II**) also crystallizes in space group *P*2_1_/*n* and is isostructural with (**I**). The asymmetric unit of [TPS]_2_[CdCl_4_] has two independent TPS^+^ cations and one [CdCl_4_]^2–^ anion (Fig. 2 ▸). The sulfonium groups are again distorted trigonal–pyramidal. For the first sulfonium (S1) cation, the S—C distances range from 1.784 (2) to 1.794 (3) Å and C—S—C angles from 103.29 (12) to 105.24 (12)°. The second sulfonium (S2) exhibits S—C bond lengths of 1.788 (2)–1.794 (3) Å with C—S—C angles spanning 100.63 (11)–106.56 (11)°. The [CdCl_4_]^2−^ anion is tetra­hedral but shows longer Cd—Cl bonds [2.4386 (6)–2.4653 (6) Å] relative to the Zn analogue, consistent with the larger ionic radius of Cd. The Cl—Cd—Cl angles vary from 103.71 (2) to 113.21 (2)°.

**Figure 3:**
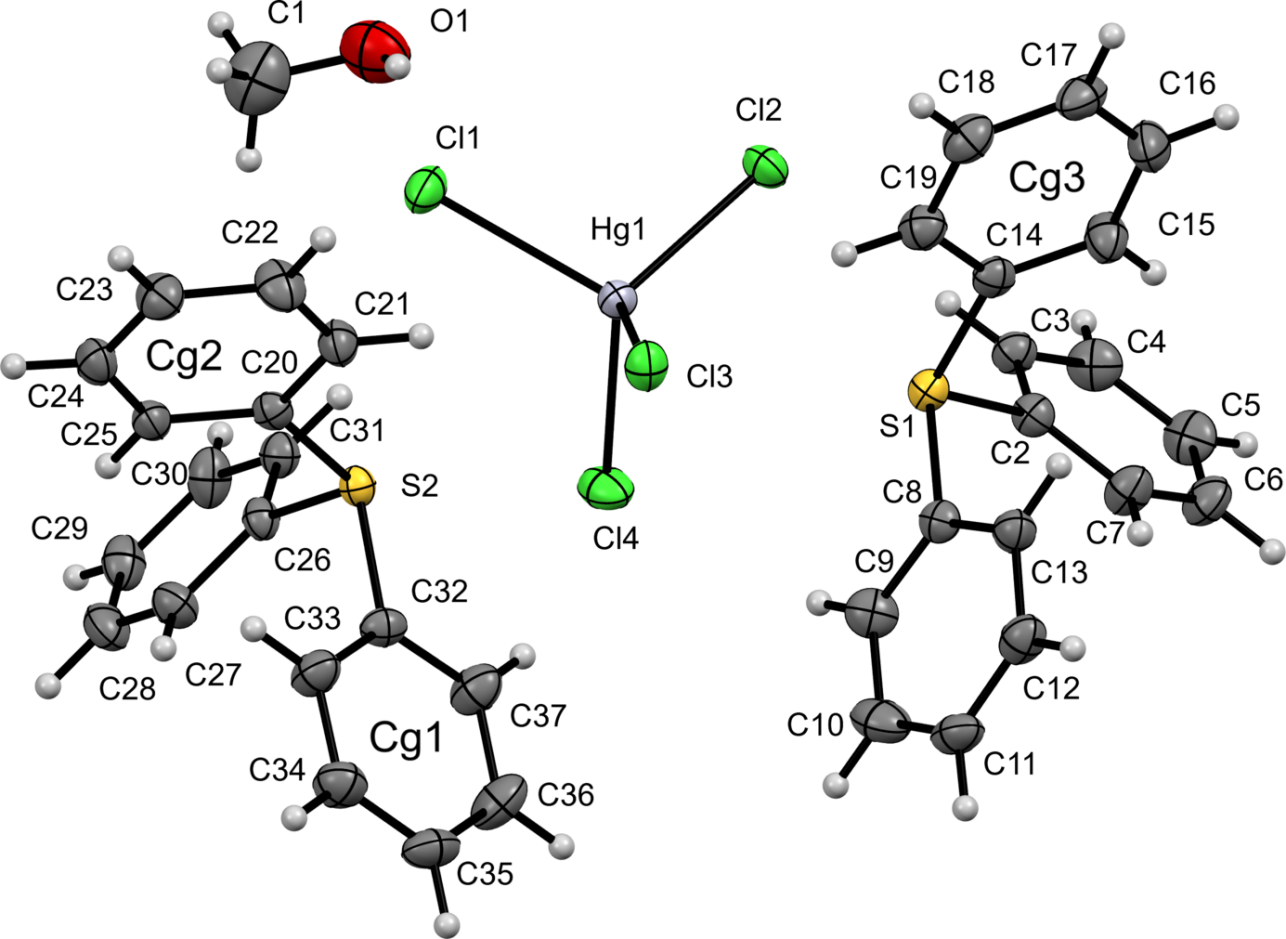
The mol­ecular structure of (**III**) with displacement ellipsoids drawn at the 50% probability level.

Compound (**III**), likewise crystallizes in the monoclinic space group *P*2_1_/*n* and in this case the asymmetric unit contains two TPS^+^ cations, one [HgCl_4_]^2–^ anion, and one methanol solvent mol­ecule (Fig. 3 ▸). The sulfonium centers retain their usual distorted trigonal–pyramidal geometries. For S1, the S—C distances lie in the 1.789 (4)–1.796 (4) Å range, with C—S—C angles of 102.6 (2)–105.5 (2)°; for S2 they span 1.783 (4)–1.784 (4) Å, with angles of 102.9 (2)–106.8 (2)°. The [HgCl_4_]^2–^ anion adopts a distorted tetra­hedral coordination with Hg–Cl distances of 2.4602 (10)–2.5333 (11) Å and Cl—Hg—Cl angles ranging from 101.77 (4) to 117.50 (4)°, having a slightly larger spread in bond angles consistent with the heavier metal center.

Comparisons among the Zn^2+^, Cd^2+^, and Hg^2+^ analogues show the systematic increase in M—Cl bond distances from Zn^2+^ to Hg^2+^, consistent with the increasing ionic radii. Nevertheless, the TPS^+^ cations exhibit only minor structural variations across the three salts, with S—C bond lengths and C—S—C angles remaining fairly constant.

## 3. Supra­molecular features

**Figure 4:**
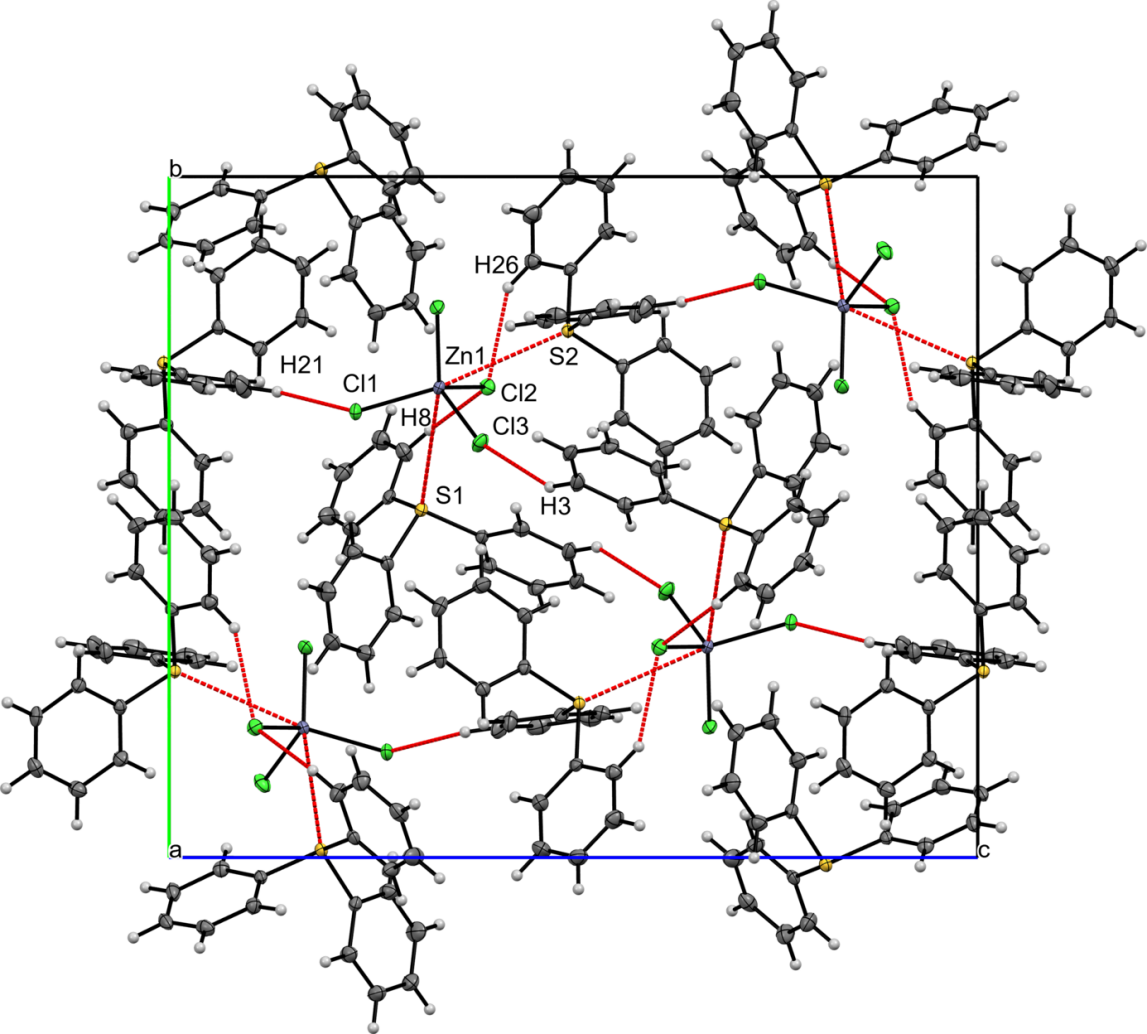
A view along the *a*-axis direction of the crystal packing of (**I**) with close contacts shown as red dashed lines.

Figs. 4 ▸, 5 ▸ and 6 ▸ illustrate the crystal packings of compounds (**I**), (**II**), and (**III**), respectively. In all three structures, the packing is consolidated by van der Waals and electrostatic inter­actions, as well as π–π stacking. Hirshfeld surfaces were generated in *Crystal Explorer 21* (Spackman *et al.*, 2021 ▸) for each crystallographically independent tri­phenyl­sulfonium (TPS) cation and for the [*M*Cl_4_]^2–^ anion (*M* = Zn^2+^, Cd^2+^, Hg^2+^). The corresponding two-dimensional fingerprint plots (McKinnon *et al.*, 2007 ▸) were analyzed to qu­antify the relative contributions of the various inter­molecular contacts (Table 1 ▸). Hydrogen bonds for (**I**), (**II**) and (**III**) are listed in Tables 2 ▸–4 ▸ ▸, respectively.

In the crystal structure of compound (**I**), two TPS^+^ cations (TPS1 and TPS2) occur in the asymmetric unit. On the Hirshfeld surfaces of TPS1 and TPS2, H⋯H inter­actions dominate, accounting for 53.7% (TPS1) and 48.8% (TPS2), followed by H⋯C/C⋯H contacts at 21.8% (TPS1) and 31.7% (TPS2). The C⋯C contacts are minor (5.6% for TPS1; 3.8% for TPS2). Notably, H⋯Cl/Cl⋯H contacts (15.5% for TPS1; 13.7% for TPS2) reflect hydrogen-bond-like inter­actions with the [ZnCl_4_]^2–^ anion. The [ZnCl_4_]^2–^ Hirshfeld surface is dominated by H⋯Cl (89.7%), with S⋯Cl (4.8%) and S⋯Zn (1.5%) also present (Table 1 ▸). Each TPS cation is anchored to the [ZnCl_4_]^2–^ anion *via* S⋯Zn [S1⋯Zn1 = 3.7464 (6), S2⋯Zn1 = 3.6708 (6) Å] and C—H⋯Cl [H26⋯Cl2 = 2.66, H8⋯Cl2 = 2.66 Å] contacts, forming discrete (TPS)_2_–ZnCl_4_ complexes. These complexes are further stitched into layers by C—H⋯Cl inter­actions, including C3—H3⋯Cl3(1 − *x*, 1 − *y*, 1 − *z*) at 2.6815 (5) Å and C21—H21⋯Cl1(−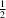 + *x*, 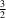 − *y*, 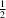 + *z*) at 2.6491 (5) Å, these layers are aligned parallel to the (101) plane (Fig. 4 ▸). All of these C—H⋯Cl short contacts can be regarded as weak hydrogen bonds (Steiner *et al.*, 1998 ▸). There is also a single inversion-centered π–π stacking inter­action [*Cg*1⋯*Cg*1(2 − *x*, 1 − *y*, 1 − *z*; centroid–centroid separation = 3.6871 (15) Å, shift = 1.471 (3) Å; *Cg*1 is the centroid of the C1–C6 ring].

**Figure 5:**
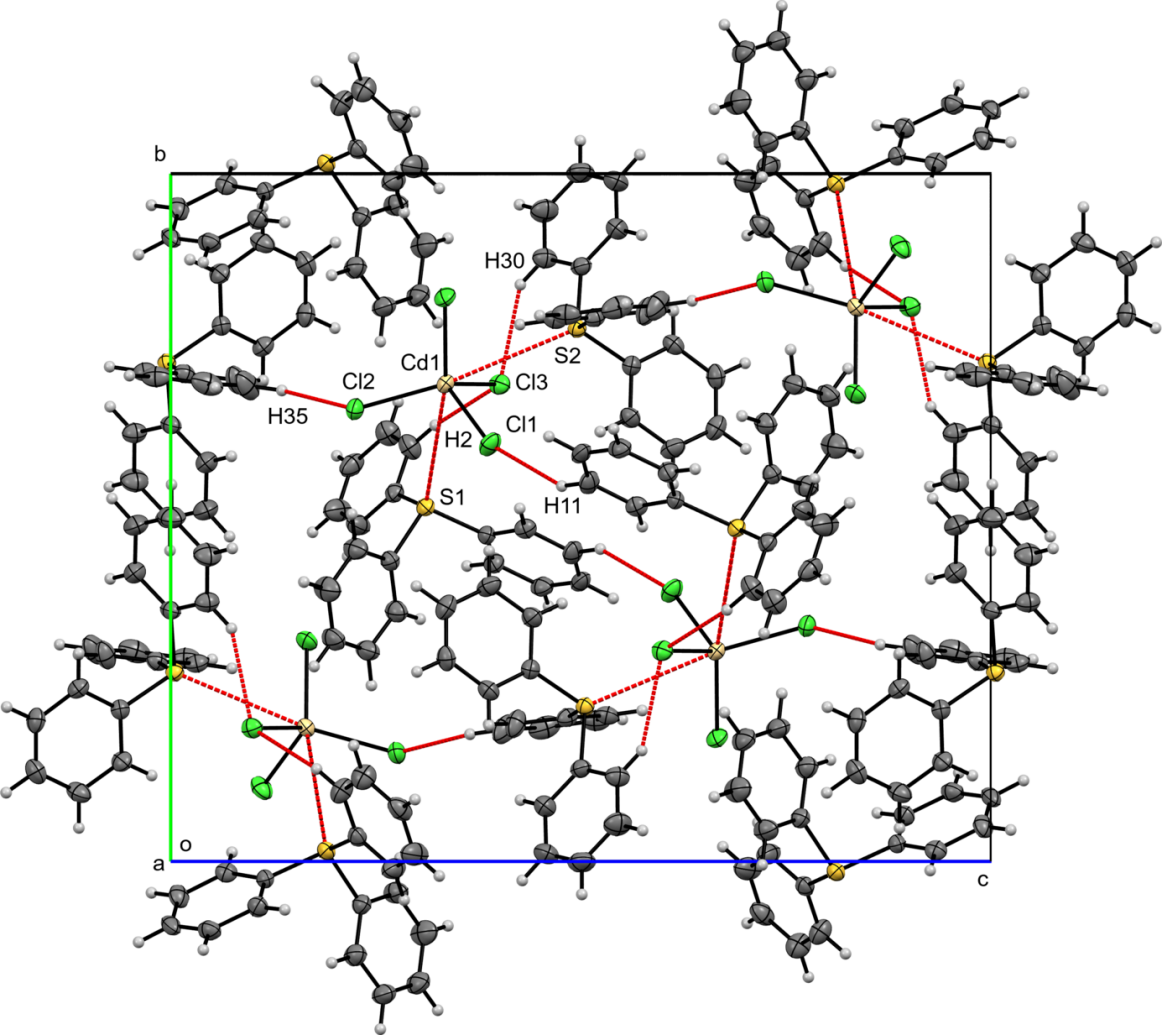
A view along the *a*-axis direction of the crystal packing of (**II**) with close contacts shown as red dashed lines.

In the crystal structure of compound (**II**), as in (**I**), two independent TPS^+^ cations (TPS1, TPS2) occur in the asymmetric unit. H⋯H inter­actions dominate (53.4% for TPS1; 48.6% for TPS2) the Hirshfeld surface, followed by H⋯C/C⋯H (21.4% for TPS1; 31.0% for TPS2), with minor C⋯C contacts (5.3% for TPS1; 3.7% for TPS2). Hydrogen-bond-like inter­actions with [CdCl_4_]^2–^ appear as H⋯Cl/Cl⋯H contributions of 16.1% (TPS1) and 14.5% (TPS2). On the anion Hirshfeld surface, H⋯Cl/Cl⋯H inter­actions dominate (88.8%), with S⋯Cl (4.2%) and S⋯Cd (1.8%) also being observed. Each TPS cation is anchored to the [CdCl_4_]^2–^ anion *via* S⋯Cd [S1⋯Cd1 = 3.8080 (6), S2⋯Cd1 = 3.7067 (6) Å] and C—H⋯Cl [H2⋯Cl3 = 2.65, H30⋯Cl3 = 2.67 Å] contacts, forming discrete (TPS)_2_–CdCl_4_ complexes. Additional short contacts [H11⋯Cl1(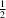 + *x*, 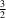 − *y*, −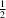 + *z*) at 2.6536 (7) Å and H35⋯Cl2(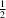 − *x*, 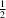 + *y*, 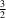 − *z*) = 2.6268 (7) Å] link these complexes into layers that are aligned parallel to the (101) plane (Fig. 5 ▸). Collectively, the S⋯Cd and C—H⋯Cl inter­actions yield a robust supra­molecular network. A single inversion-centered π–π stacking inter­action [*Cg*1⋯*Cg*(1 − *x*, 1 − *y*, 1 − *z*; centroid–centroid = 3.736 (2) Å, shift = 1.553 (4) Å; *Cg*1 is the centroid of the C7–C12 ring] is also observed.

**Figure 6:**
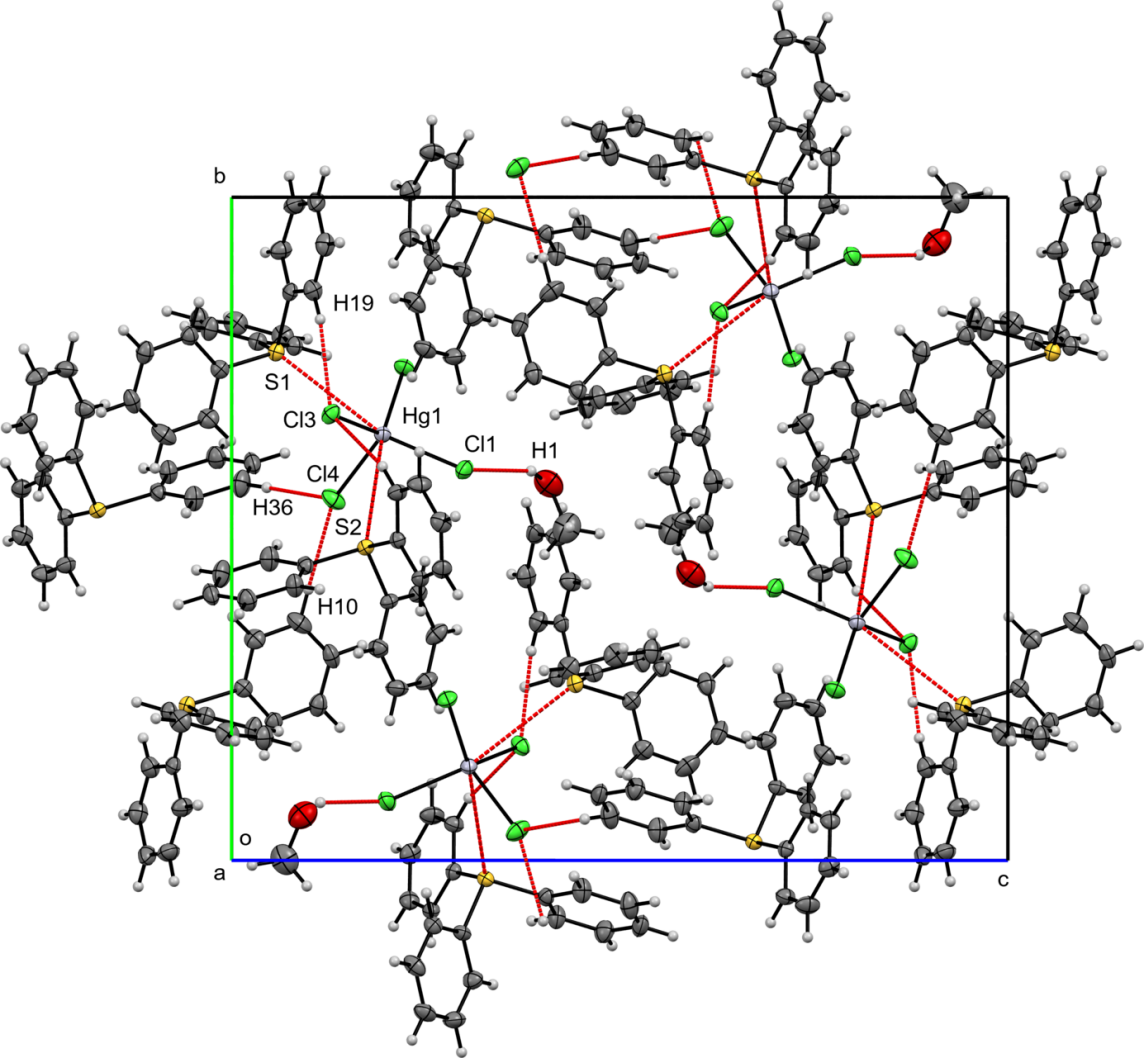
A view along the *a*-axis direction of the crystal packing of (**III**) with close contacts shown as red dashed lines.

In the crystal structure of compound (**III**), two independent TPS^+^ cations are present. H⋯H contacts dominate the Hirshfeld surfaces of both (56.8% for TPS1; 51.4% for TPS2). The H⋯C contacts account for 21.9% (TPS1) and 19.5% (TPS2), while C⋯C contacts are slightly higher for TPS2 (8.2%) than for TPS1 (5.3%). Contacts with the [HgCl_4_]^2–^ anion, include H⋯Cl (11.0% for TPS1; 16.2% for TPS2) and S⋯Cl (1.1% and 1.2%, respectively). A small but non-negligible H⋯O contribution (2.7% for TPS1; 0.7% for TPS2) arises from the solvated methanol mol­ecule. On the [HgCl_4_]^2–^ Hirshfeld surface, H⋯Cl contacts dominate (88.1%), with S⋯Cl (4.2%) and S⋯Hg (2.0%) also being present. Each TPS cation binds the [HgCl_4_]^2–^ anion *via* S⋯Hg [Hg1⋯S1 = 3.7674 (12), Hg1⋯S2 = 3.8233 (10) Å] and C—H⋯Cl inter­actions [Cl3⋯H19 = 2.64, Cl3⋯H21 = 2.72 Å], forming (TPS)_2_–HgCl_4_ complexes. Unlike in (**I**) and (**II**), two such complexes associate to form (TPS)_2_–HgCl_4_ dimers *via* additional C—H⋯Cl contacts [Cl4⋯H36 = 2.6231 (13) Å, symmetry code: (1 − *x*, 1 − *y*, 2 − *z*); Cl4⋯H10 = 2.6530 (13) Å, symmetry code: (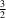 − *x*, 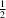 + *y*, 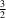 − *z*)], with neighboring dimers inter­act only weakly (Fig. 6 ▸). These dimers also incorporate a hydrogen-bonded methanol mol­ecule [O1⋯Cl1 = 3.188 (5) Å] (Table 4 ▸). An inversion-centered π–π stacking inter­action is observed in (**III**) [*Cg*1⋯*Cg*1(2 − *x*, 1 − *y*, 2 − *z*; centroid–centroid = 3.658 (4) Å, shift = 1.054 (8) Å; *Cg*1 is the centroid of the C32–C37 ring] is accompanied by a second π–π inter­action [*Cg*2⋯*Cg*3(2 − *x*, 1 − *y*, 1 − *z*; angle = 7.464 (14)°, centroid–centroid = 3.910 (3) Å, shift = 2.097 (6) Å; *Cg*1 and*Cg*2 are the centroids of the C20–C25 and C14–C19 rings, respectively].

## 4. Database survey

A search of the web-based Cambridge Structural Database (CSD, website, accessed on January 16, 2025; Groom *et al.*, 2016 ▸) for the tri­phenyl­sulfonium ion yielded 24 entries with the majority (19) being TPS^+^ complexes. In the search, two of the returns were imine, one was a thia­zine motif and two are nitrile derivatives of tri­phenyl­sulfonium. Simple salts derivatives of TPS^+^ include the bis­[(tri­fluoro­meth­yl)sulfon­yl]aza­dine salt (CSD refcode BANYOH; Siu *et al.*, 2017 ▸), azide (FOYKEK; Klapötke *et al.*, 2009*a* ▸), tri­fluoro­methansulfonate (LECWOI; Zhang *et al.*, 2017 ▸), chloride monohydrate (NIMMIJ; Luo *et al.*, 2022*a* ▸), bromide hydrate (ROKYAS; Klapötke *et al.*, 2009*a* ▸), tetra­fluoro­borate (TUBXET; Ovchinnikov *et al.*, 1996 ▸) and the recently reported triiodide (FUMMEJ; Artis *et al.*, 2025 ▸), perchlorate (FUMMIN; Artis *et al.*, 2025 ▸) and hexa­fluoro­phosphate (FUMMOT; Artis *et al.*, 2025 ▸) salts.

Metal-based anionic salts of the formula [TPS]_2_*M*Cl_*x*_ (where *x* = 5 or 6) include the bis­(tri­phenyl­sulfonium) penta­chloro­anti­mony(III) (MUFFAY; Liao *et al.* 2024 ▸) and its aceto­nitrile solvate (MUFFIG; Liao *et al.* 2024 ▸), the bis­(tri­phenyl­sulfonium) hexa­chloro­tin(IV) (NIMMAB; Luo *et al.*, 2022*a* ▸), and bis­(tri­phenyl­sulfonium) hexa­chloro­tellurium(V) (NIMMEF; Luo *et al.*, 2022*a* ▸). Finally more unique silver and manganese structures are reported, the bis­(μ_2_-1,3-azido)­silver(I) (QOSQEV; Klapötke *et al.*, 2009*b* ▸) and the tris­(μ_2_-dicyanamido)­manganese(II) (SABFUX; Schlueter *et al.*, 2004 ▸) structures with tri­phenyl­sulfonium.

## 5. Synthesis and crystallization

Compound (**I**) was synthesized by dissolving tri­phenyl­sulfonium chloride (0.0947g, 0.317 mmol, purchased from TCI America) in 20 ml of methanol within a glass beaker before adding zinc(II) chloride (0.0216 g, 0.158 mmol, purchased from Millipore Sigma) to the reaction mixture. The reaction was covered with a watch glass before heating and stirring until complete dissolution. After dissolving, the reaction vessel was covered in parafilm with small holes to allow for cooling and evaporation over the course of one week. The resulting crystals in the form of colorless irregular blocks were washed with diethyl ether in pre-weighed crucibles during vacuum filtration, and reweighed once crucibles were dried. Yield, 0.0961g (82.6%). Selected IR bands (ATR-IR cm^−1^): 3100(*w*), 3050(*w*), 3000(*w*), 1750(*w*), 1600(*w*), 1550(*s*), 1450(*s*), 1250(*w*), 1150(*w*), 1000(*w*), 750(*s*), 650(*s*), 500(*s*).

Compound (**II**) was synthesized by dissolving CdCl_2_ (0.0382g, 0.167 mmol, purchased from Baker and Adamson) in 100 ml of methanol and heating to a boil to dissolved. Tri­phenyl­sulfonium chloride (0.100 g, 0.335 mmol, purchased from TCI America) was added in one portion and the solution was refluxed for 10 minutes to ensure complete dissolution. The reaction vessel was covered in parafilm to allow for evaporation to produce X-ray quality crystals in the form of colorless irregular blocks. Yield, 0.0845g (61.1%). Selected IR bands (ATR-IR cm^−1^): 3108(*w*), 1524(*m*), 1502(*m*), 1096(*w*), 998(*w*), 820(*s*), 804(*s*), 752(*s*), 730(*s*).

Compound (**III**) was prepared by dissolving HgCl_2_ (0.0454 g, 0.167 mmol, purchased from Reagents, Inc.) in 50 ml of methanol at room temperature. Tri­phenyl­sulfonium chloride (0.100 g, 0.335 mmol, purchased from TCI America) was added in one portion and the solution was stirred for 10 minutes. The reaction vessel was covered in parafilm to allow for evaporation to produce X-ray quality crystals as colorless irregular blocks. Yield, 0.1020 g (70.3%) Selected IR Bands (ATR-IRcm^−1^): 3098 (*w*), 1738(*s*), 1473(*s*), 1456(*s*), 1369(*w*), 1228(*m*), 1055(*w*), 991(*s*), 862(*s*), 825(*s*), 753(*s*), 679(*s*).

## 6. Refinement

Crystal data, data collection and structure refinement details are summarized in Table 5 ▸. All carbon-bound H atoms were positioned geometrically and refined as riding atoms: C—H = 0.95–0.98 Å with *U*_iso_(H) = 1.2*U*_eq_(C). The methanol O-bonded H atom in (**III**) was geometrically placed (O—H = 0.84 Å) and refined as riding.

## Supplementary Material

Crystal structure: contains datablock(s) global, II, III, I. DOI: 10.1107/S2056989025002245/hb8121sup1.cif

Structure factors: contains datablock(s) I. DOI: 10.1107/S2056989025002245/hb8121Isup2.hkl

Structure factors: contains datablock(s) II. DOI: 10.1107/S2056989025002245/hb8121IIsup3.hkl

Structure factors: contains datablock(s) III. DOI: 10.1107/S2056989025002245/hb8121IIIsup4.hkl

CCDC references: 2430866, 2430865, 2430864

Additional supporting information:  crystallographic information; 3D view; checkCIF report

## Figures and Tables

**Table 1:** Contributions of selected inter­molecular contacts (%)

Contact	(**I**) (TPS1)	(**I**) (TPS2)	(**I**) (ZnCl_4_)	(**II**) (TPS1)	(**II**) (TPS2)	(**II**) (CdCl_4_)	(**III**) (TPS1)	(**III**) (TPS2)	(**III**) (HgCl_4_)
C⋯C	5.6	3.8	–	5.3	3.7	–	5.3	8.2	–
H⋯C/C⋯H	21.8	31.7	–	21.4	31.0	–	21.9	19.5	–
H⋯H	53.7	48.8	–	53.4	48.6	–	56.8	51.4	–
H⋯Cl/Cl⋯H	15.5	13.7	89.7	16.1	14.5	88.8	11.0	16.2	88.1
S⋯Cl	1.2	1.3	4.8	1.1	1.2	4.2	1.1	1.2	4.2
H⋯O/O⋯H	–	–	–	–	–	–	2.7	0.7	–
S⋯*M*	–	–	1.5	–	–	1.8	–	–	2.0

**Table 2:** Hydrogen-bond geometry (Å, °) for (**I**)

*D*—H⋯*A*	*D*—H	H⋯*A*	*D*⋯*A*	*D*—H⋯*A*
C3—H3⋯Cl3^i^	0.95	2.68	3.5210 (19)	148
C8—H8⋯Cl2	0.95	2.66	3.552 (2)	158
C14—H14⋯Cl1	0.95	2.75	3.610 (2)	151
C21—H21⋯Cl1^ii^	0.95	2.65	3.554 (2)	159
C24—H24⋯Cl4	0.95	2.74	3.5792 (19)	147
C26—H26⋯Cl2	0.95	2.66	3.509 (2)	149
C33—H33⋯Cl3^i^	0.95	2.76	3.695 (2)	167
C35—H35⋯Cl1^iii^	0.95	2.81	3.592 (2)	140

Symmetry codes: (i) 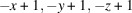; (ii) 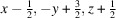; (iii) 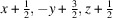.

**Table 3:** Hydrogen-bond geometry (Å, °) for (**II**)

*D*—H⋯*A*	*D*—H	H⋯*A*	*D*⋯*A*	*D*—H⋯*A*
C2—H2⋯Cl3^i^	0.95	2.65	3.561 (3)	162
C5—H5⋯Cl4	0.95	2.81	3.453 (3)	126
C11—H11⋯Cl1^ii^	0.95	2.65	3.512 (3)	150
C18—H18⋯Cl2^i^	0.95	2.73	3.593 (3)	152
C21—H21⋯Cl2^iii^	0.95	2.80	3.559 (3)	137
C23—H23⋯Cl1^i^	0.95	2.74	3.667 (3)	165
C30—H30⋯Cl3^ii^	0.95	2.67	3.528 (3)	150
C32—H32⋯Cl4^ii^	0.95	2.76	3.588 (3)	146
C35—H35⋯Cl2	0.95	2.63	3.508 (3)	155

Symmetry codes: (i) 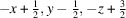; (ii) 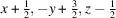; (iii) 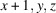.

**Table 4:** Hydrogen-bond geometry (Å, °) for (**III**)

*D*—H⋯*A*	*D*—H	H⋯*A*	*D*⋯*A*	*D*—H⋯*A*
O1—H1⋯Cl1	0.84	2.41	3.187 (5)	155
C3—H3⋯Cl2^i^	0.95	2.75	3.631 (4)	155
C10—H10⋯Cl4^ii^	0.95	2.65	3.588 (6)	168
C13—H13⋯O1^iii^	0.95	2.56	3.192 (7)	124
C19—H19⋯Cl3^i^	0.95	2.64	3.542 (5)	159
C21—H21⋯Cl3	0.95	2.72	3.639 (4)	163
C31—H31⋯Cl1	0.95	2.81	3.630 (5)	145
C36—H36⋯Cl4^iv^	0.95	2.62	3.573 (5)	178
C37—H37⋯Cl4	0.95	2.81	3.712 (5)	159

Symmetry codes: (i) 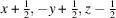; (ii) 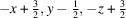; (iii) 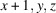; (iv) 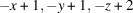.

**Table 5:** Experimental details

	(**I**)	(**II**)	(**III**)
Crystal data			
Chemical formula	(C_18_H_15_S)_2_[ZnCl_4_]	(C_18_H_15_S)_2_[CdCl_4_]	(C_18_H_15_S)_2_[HgCl_4_]·CH_4_O
*M*_r_	733.89	780.92	901.15
Crystal system, space group	Monoclinic, *P*2_1_/*n*	Monoclinic, *P*2_1_/*n*	Monoclinic, *P*2_1_/*n*
Temperature (K)	100	100	100
*a*, *b*, *c* (Å)	9.1630 (1), 17.5749 (2), 21.1697 (2)	9.2696 (1), 17.6682 (2), 21.4072 (2)	9.43577 (14), 18.0709 (3), 21.2467 (3)
β (°)	99.322 (1)	99.801 (1)	95.1778 (14)
*V* (Å^3^)	3364.12 (6)	3454.84 (6)	3608.05 (9)
*Z*	4	4	4
Radiation type	Cu *K*α	Cu *K*α	Cu *K*α
μ (mm^−1^)	5.29	9.22	11.68
Crystal size (mm)	0.41 × 0.29 × 0.16	0.19 × 0.13 × 0.09	0.11 × 0.06 × 0.05

Data collection			
Diffractometer	XtaLAB Synergy, Single source at home/near, HyPix3000	XtaLAB Synergy, Single source at home/near, HyPix3000	XtaLAB Synergy, Single source at home/near, HyPix3000
Absorption correction	Gaussian (*CrysAlis PRO*; Rigaku OD, 2023 ▸)	Multi-scan (*CrysAlis PRO*; Rigaku OD, 2023 ▸)	Multi-scan (*CrysAlis PRO*; Rigaku OD, 2023 ▸)
*T*_min_, *T*_max_	0.398, 1.000	0.488, 1.000	0.644, 1.000
No. of measured, independent and observed [*I* > 2σ(*I*)] reflections	19982, 6146, 5660	20400, 6456, 5793	22094, 6743, 6142
*R*_int_	0.036	0.055	0.054
(sin θ/λ)_max_ (Å^−1^)	0.602	0.609	0.610

Refinement			
*R*[*F*^2^ > 2σ(*F*^2^)], *wR*(*F*^2^), *S*	0.029, 0.074, 1.04	0.026, 0.066, 1.05	0.031, 0.080, 1.07
No. of reflections	6146	6456	6743
No. of parameters	388	388	408
H-atom treatment	H-atom parameters constrained	H-atom parameters constrained	H-atom parameters constrained
Δρ_max_, Δρ_min_ (e Å^−3^)	0.32, −0.37	0.63, −0.49	2.45, −2.10

Computer programs: *CrysAlis PRO* (Rigaku OD, 2023 ▸), *SHELXT2018/2* (Sheldrick, 2015*a* ▸), *SHELXL2018/3* (Sheldrick, 2015*b* ▸) and *OLEX2* (Dolomanov *et al.*, 2009 ▸).

## supplementary crystallographic information

### Bis(triphenylsulfonium) tetrachloridozinc(II) (I) Crystal data

**Table:**

(C_18_H_15_S)_2_[ZnCl_4_]	*F*(000) = 1504
*M**_r_* = 733.89	*D*_x_ = 1.449 Mg m^−^^3^
Monoclinic, *P*2_1_/*n*	Cu *K*α radiation, λ = 1.54184 Å
*a* = 9.1630 (1) Å	Cell parameters from 13744 reflections
*b* = 17.5749 (2) Å	θ = 2.1–69.5°
*c* = 21.1697 (2) Å	µ = 5.29 mm^−^^1^
β = 99.322 (1)°	*T* = 100 K
*V* = 3364.12 (6) Å^3^	Irregular, clear colourless
*Z* = 4	0.41 × 0.29 × 0.16 mm

### Bis(triphenylsulfonium) tetrachloridozinc(II) (I) Data collection

**Table:**

XtaLAB Synergy, Single source at home/near, HyPix3000 diffractometer	6146 independent reflections
Radiation source: micro-focus sealed X-ray tube, PhotonJet (Cu) X-ray Source	5660 reflections with *I* > 2σ(*I*)
Mirror monochromator	*R*_int_ = 0.036
Detector resolution: 10.0000 pixels mm^-1^	θ_max_ = 68.2°, θ_min_ = 3.3°
ω scans	*h* = −11→10
Absorption correction: gaussian (CrysAlisPro; Rigaku OD, 2023)	*k* = −21→20
*T*_min_ = 0.398, *T*_max_ = 1.000	*l* = −25→25
19982 measured reflections	

### Bis(triphenylsulfonium) tetrachloridozinc(II) (I) Refinement

**Table:**

Refinement on *F*^2^	Primary atom site location: dual
Least-squares matrix: full	Hydrogen site location: inferred from neighbouring sites
*R*[*F*^2^ > 2σ(*F*^2^)] = 0.029	H-atom parameters constrained
*wR*(*F*^2^) = 0.074	*w* = 1/[σ^2^(*F*_o_^2^) + (0.0352*P*)^2^ + 1.5636*P*] where *P* = (*F*_o_^2^ + 2*F*_c_^2^)/3
*S* = 1.04	(Δ/σ)_max_ = 0.001
6146 reflections	Δρ_max_ = 0.32 e Å^−^^3^
388 parameters	Δρ_min_ = −0.37 e Å^−^^3^
0 restraints	

### Bis(triphenylsulfonium) tetrachloridozinc(II) (I) Special details

**Table:**

Geometry. All esds (except the esd in the dihedral angle between two l.s. planes) are estimated using the full covariance matrix. The cell esds are taken into account individually in the estimation of esds in distances, angles and torsion angles; correlations between esds in cell parameters are only used when they are defined by crystal symmetry. An approximate (isotropic) treatment of cell esds is used for estimating esds involving l.s. planes.

### Bis(triphenylsulfonium) tetrachloridozinc(II) (I) Fractional atomic coordinates and isotropic or equivalent isotropic displacement parameters (Å^2^)

**Table:**

	*x*	*y*	*z*	*U*_iso_*/*U*_eq_	
C1	0.8253 (2)	0.47093 (10)	0.38690 (8)	0.0146 (4)	
S1	0.74640 (5)	0.51070 (3)	0.31186 (2)	0.01438 (10)	
C2	0.7441 (2)	0.48286 (11)	0.43563 (9)	0.0184 (4)	
H2	0.657200	0.513262	0.428924	0.022*	
C3	0.7930 (2)	0.44910 (12)	0.49490 (9)	0.0231 (4)	
H3	0.738618	0.455906	0.529078	0.028*	
C4	0.9207 (2)	0.40567 (11)	0.50395 (9)	0.0225 (4)	
H4	0.952540	0.382096	0.544203	0.027*	
C5	1.0025 (2)	0.39624 (11)	0.45497 (10)	0.0225 (4)	
H5	1.091095	0.367214	0.462098	0.027*	
C6	0.9557 (2)	0.42893 (11)	0.39561 (9)	0.0195 (4)	
H6	1.011171	0.422818	0.361730	0.023*	
C7	0.9009 (2)	0.52951 (11)	0.27204 (9)	0.0165 (4)	
C8	0.9798 (2)	0.59510 (12)	0.29067 (10)	0.0236 (4)	
H8	0.950942	0.627520	0.322368	0.028*	
C9	1.1020 (2)	0.61272 (13)	0.26219 (10)	0.0253 (5)	
H9	1.158056	0.657224	0.274808	0.030*	
C10	1.1421 (2)	0.56588 (12)	0.21575 (10)	0.0247 (5)	
H10	1.225365	0.578253	0.196228	0.030*	
C11	1.0618 (3)	0.50129 (13)	0.19762 (11)	0.0345 (6)	
H11	1.090248	0.469337	0.165533	0.041*	
C12	0.9398 (3)	0.48194 (12)	0.22537 (10)	0.0272 (5)	
H12	0.884440	0.437234	0.212702	0.033*	
C13	0.6535 (2)	0.43173 (11)	0.26970 (8)	0.0166 (4)	
C14	0.5248 (2)	0.44948 (12)	0.22839 (10)	0.0235 (4)	
H14	0.490734	0.500518	0.223418	0.028*	
C15	0.4469 (2)	0.39056 (13)	0.19437 (11)	0.0301 (5)	
H15	0.357985	0.401338	0.165974	0.036*	
C16	0.4974 (2)	0.31665 (12)	0.20143 (10)	0.0256 (5)	
H16	0.443526	0.276913	0.177755	0.031*	
C17	0.6264 (2)	0.30022 (12)	0.24291 (10)	0.0259 (5)	
H17	0.660949	0.249241	0.247467	0.031*	
C18	0.7052 (2)	0.35761 (12)	0.27774 (10)	0.0235 (4)	
H18	0.793156	0.346507	0.306655	0.028*	
S2	0.53980 (5)	0.77362 (2)	0.49292 (2)	0.01309 (10)	
C19	0.3622 (2)	0.79066 (11)	0.51477 (9)	0.0164 (4)	
C20	0.3423 (2)	0.80035 (13)	0.57820 (10)	0.0257 (5)	
H20	0.424629	0.801209	0.611844	0.031*	
C21	0.1995 (3)	0.80870 (13)	0.59095 (11)	0.0305 (5)	
H21	0.183467	0.815984	0.633755	0.037*	
C22	0.0806 (2)	0.80650 (12)	0.54183 (11)	0.0275 (5)	
H22	−0.016761	0.812408	0.551145	0.033*	
C23	0.1015 (2)	0.79579 (12)	0.47909 (11)	0.0240 (4)	
H23	0.018640	0.793888	0.445736	0.029*	
C24	0.2437 (2)	0.78781 (11)	0.46489 (9)	0.0183 (4)	
H24	0.259268	0.780567	0.422012	0.022*	
C25	0.6307 (2)	0.86388 (10)	0.49645 (9)	0.0154 (4)	
C26	0.7194 (2)	0.87534 (12)	0.44999 (9)	0.0200 (4)	
H26	0.728879	0.836998	0.419279	0.024*	
C27	0.7940 (2)	0.94416 (12)	0.44938 (10)	0.0260 (5)	
H27	0.855443	0.953012	0.418043	0.031*	
C28	0.7792 (2)	0.99994 (12)	0.49421 (11)	0.0286 (5)	
H28	0.831311	1.046631	0.493807	0.034*	
C29	0.6889 (3)	0.98766 (12)	0.53940 (10)	0.0296 (5)	
H29	0.678724	1.026266	0.569799	0.035*	
C30	0.6129 (2)	0.91971 (11)	0.54101 (9)	0.0241 (4)	
H30	0.550007	0.911500	0.571895	0.029*	
C31	0.6273 (2)	0.72101 (11)	0.56086 (8)	0.0143 (4)	
C32	0.5861 (2)	0.64505 (11)	0.56004 (9)	0.0196 (4)	
H32	0.524944	0.624018	0.523704	0.023*	
C33	0.6357 (2)	0.60016 (12)	0.61330 (10)	0.0231 (4)	
H33	0.607100	0.548260	0.614129	0.028*	
C34	0.7274 (2)	0.63185 (12)	0.66521 (9)	0.0224 (4)	
H34	0.760331	0.601393	0.701813	0.027*	
C35	0.7715 (2)	0.70692 (12)	0.66456 (9)	0.0211 (4)	
H35	0.836093	0.727328	0.700148	0.025*	
C36	0.7215 (2)	0.75270 (11)	0.61196 (9)	0.0179 (4)	
H36	0.751214	0.804423	0.611030	0.021*	
Cl1	0.54216 (5)	0.65468 (3)	0.23073 (2)	0.02032 (11)	
Zn1	0.54009 (3)	0.69024 (2)	0.33396 (2)	0.01294 (7)	
Cl2	0.77135 (5)	0.69108 (3)	0.39384 (2)	0.01820 (11)	
Cl3	0.40705 (5)	0.60886 (3)	0.38479 (2)	0.02266 (11)	
Cl4	0.43578 (5)	0.80813 (3)	0.33149 (2)	0.01928 (11)	

### Bis(triphenylsulfonium) tetrachloridozinc(II) (I) Atomic displacement parameters (Å^2^)

**Table:**

	*U*^11^	*U*^22^	*U*^33^	*U*^12^	*U*^13^	*U*^23^
C1	0.0172 (9)	0.0137 (9)	0.0119 (9)	−0.0019 (7)	−0.0006 (7)	0.0015 (7)
S1	0.0168 (2)	0.0139 (2)	0.0117 (2)	0.00158 (17)	0.00013 (17)	0.00038 (17)
C2	0.0164 (9)	0.0197 (10)	0.0189 (10)	−0.0037 (8)	0.0024 (8)	−0.0005 (8)
C3	0.0297 (11)	0.0252 (11)	0.0143 (9)	−0.0090 (9)	0.0035 (8)	0.0024 (8)
C4	0.0291 (11)	0.0193 (10)	0.0161 (10)	−0.0076 (9)	−0.0054 (8)	0.0056 (8)
C5	0.0212 (10)	0.0183 (10)	0.0248 (11)	0.0019 (8)	−0.0059 (8)	0.0035 (8)
C6	0.0207 (10)	0.0203 (10)	0.0168 (9)	0.0012 (8)	0.0006 (8)	−0.0011 (8)
C7	0.0202 (9)	0.0171 (9)	0.0115 (9)	0.0028 (8)	0.0008 (7)	0.0040 (7)
C8	0.0248 (10)	0.0264 (11)	0.0199 (10)	−0.0030 (9)	0.0048 (8)	−0.0063 (9)
C9	0.0223 (10)	0.0290 (12)	0.0238 (11)	−0.0049 (9)	0.0011 (8)	−0.0010 (9)
C10	0.0240 (11)	0.0272 (11)	0.0246 (11)	0.0064 (9)	0.0088 (9)	0.0087 (9)
C11	0.0548 (15)	0.0225 (11)	0.0332 (13)	0.0012 (11)	0.0284 (12)	−0.0003 (10)
C12	0.0439 (13)	0.0166 (10)	0.0248 (11)	−0.0042 (9)	0.0169 (10)	−0.0043 (9)
C13	0.0204 (9)	0.0173 (9)	0.0119 (9)	−0.0022 (8)	0.0018 (7)	−0.0017 (7)
C14	0.0245 (11)	0.0180 (10)	0.0256 (11)	0.0021 (8)	−0.0034 (8)	0.0001 (8)
C15	0.0270 (11)	0.0281 (12)	0.0293 (12)	0.0006 (9)	−0.0132 (9)	−0.0018 (10)
C16	0.0313 (12)	0.0221 (11)	0.0214 (11)	−0.0052 (9)	−0.0018 (9)	−0.0034 (9)
C17	0.0324 (12)	0.0181 (10)	0.0252 (11)	0.0022 (9)	−0.0015 (9)	−0.0024 (8)
C18	0.0257 (11)	0.0194 (10)	0.0223 (10)	0.0034 (9)	−0.0057 (8)	−0.0007 (8)
S2	0.0147 (2)	0.0147 (2)	0.0096 (2)	−0.00055 (17)	0.00127 (16)	−0.00027 (16)
C19	0.0181 (9)	0.0157 (9)	0.0164 (9)	0.0016 (8)	0.0053 (7)	0.0025 (8)
C20	0.0291 (11)	0.0332 (12)	0.0155 (10)	0.0066 (9)	0.0055 (8)	0.0030 (9)
C21	0.0363 (12)	0.0359 (13)	0.0237 (11)	0.0099 (10)	0.0185 (10)	0.0078 (9)
C22	0.0219 (11)	0.0231 (11)	0.0413 (13)	0.0027 (9)	0.0167 (10)	0.0074 (9)
C23	0.0172 (10)	0.0196 (10)	0.0347 (12)	−0.0027 (8)	0.0025 (9)	−0.0011 (9)
C24	0.0194 (10)	0.0150 (9)	0.0206 (10)	−0.0006 (8)	0.0032 (8)	−0.0015 (8)
C25	0.0170 (9)	0.0126 (9)	0.0150 (9)	0.0003 (7)	−0.0018 (7)	0.0021 (7)
C26	0.0196 (10)	0.0193 (10)	0.0207 (10)	−0.0009 (8)	0.0024 (8)	−0.0006 (8)
C27	0.0269 (11)	0.0248 (11)	0.0269 (11)	−0.0043 (9)	0.0062 (9)	0.0044 (9)
C28	0.0342 (12)	0.0184 (11)	0.0300 (12)	−0.0059 (9)	−0.0044 (10)	0.0026 (9)
C29	0.0458 (14)	0.0177 (11)	0.0243 (11)	−0.0025 (10)	0.0029 (10)	−0.0052 (9)
C30	0.0351 (12)	0.0194 (10)	0.0183 (10)	−0.0008 (9)	0.0055 (9)	−0.0014 (8)
C31	0.0139 (9)	0.0163 (9)	0.0122 (9)	0.0017 (7)	0.0006 (7)	0.0010 (7)
C32	0.0206 (10)	0.0192 (10)	0.0175 (10)	−0.0036 (8)	−0.0011 (8)	−0.0012 (8)
C33	0.0245 (10)	0.0181 (10)	0.0261 (11)	−0.0019 (8)	0.0025 (8)	0.0044 (8)
C34	0.0225 (10)	0.0277 (11)	0.0170 (10)	0.0037 (9)	0.0032 (8)	0.0078 (8)
C35	0.0215 (10)	0.0283 (11)	0.0120 (9)	−0.0006 (9)	−0.0022 (8)	−0.0013 (8)
C36	0.0194 (9)	0.0183 (10)	0.0158 (9)	−0.0017 (8)	0.0026 (7)	−0.0017 (8)
Cl1	0.0317 (3)	0.0195 (2)	0.0104 (2)	0.00282 (19)	0.00520 (18)	0.00101 (17)
Zn1	0.01626 (13)	0.01306 (13)	0.00941 (13)	0.00044 (9)	0.00177 (10)	0.00183 (9)
Cl2	0.0158 (2)	0.0190 (2)	0.0185 (2)	0.00186 (17)	−0.00122 (17)	−0.00075 (17)
Cl3	0.0242 (2)	0.0237 (2)	0.0204 (2)	−0.00547 (19)	0.00445 (19)	0.00802 (19)
Cl4	0.0259 (2)	0.0164 (2)	0.0145 (2)	0.00658 (18)	0.00023 (18)	0.00183 (17)

### Bis(triphenylsulfonium) tetrachloridozinc(II) (I) Geometric parameters (Å, º)

**Table:**

C1—S1	1.7784 (18)	S2—C31	1.7868 (18)
C1—C2	1.383 (3)	C19—C20	1.394 (3)
C1—C6	1.391 (3)	C19—C24	1.388 (3)
S1—C7	1.7919 (19)	C20—H20	0.9500
S1—C13	1.7892 (19)	C20—C21	1.386 (3)
C2—H2	0.9500	C21—H21	0.9500
C2—C3	1.395 (3)	C21—C22	1.380 (3)
C3—H3	0.9500	C22—H22	0.9500
C3—C4	1.384 (3)	C22—C23	1.385 (3)
C4—H4	0.9500	C23—H23	0.9500
C4—C5	1.384 (3)	C23—C24	1.391 (3)
C5—H5	0.9500	C24—H24	0.9500
C5—C6	1.385 (3)	C25—C26	1.388 (3)
C6—H6	0.9500	C25—C30	1.389 (3)
C7—C8	1.384 (3)	C26—H26	0.9500
C7—C12	1.384 (3)	C26—C27	1.390 (3)
C8—H8	0.9500	C27—H27	0.9500
C8—C9	1.390 (3)	C27—C28	1.386 (3)
C9—H9	0.9500	C28—H28	0.9500
C9—C10	1.377 (3)	C28—C29	1.380 (3)
C10—H10	0.9500	C29—H29	0.9500
C10—C11	1.373 (3)	C29—C30	1.386 (3)
C11—H11	0.9500	C30—H30	0.9500
C11—C12	1.386 (3)	C31—C32	1.387 (3)
C12—H12	0.9500	C31—C36	1.387 (3)
C13—C14	1.385 (3)	C32—H32	0.9500
C13—C18	1.387 (3)	C32—C33	1.391 (3)
C14—H14	0.9500	C33—H33	0.9500
C14—C15	1.391 (3)	C33—C34	1.387 (3)
C15—H15	0.9500	C34—H34	0.9500
C15—C16	1.379 (3)	C34—C35	1.381 (3)
C16—H16	0.9500	C35—H35	0.9500
C16—C17	1.384 (3)	C35—C36	1.390 (3)
C17—H17	0.9500	C36—H36	0.9500
C17—C18	1.382 (3)	Cl1—Zn1	2.2760 (5)
C18—H18	0.9500	Zn1—Cl2	2.2863 (5)
S2—C19	1.7879 (19)	Zn1—Cl3	2.2615 (5)
S2—C25	1.7876 (19)	Zn1—Cl4	2.2788 (5)
			
C2—C1—S1	114.63 (14)	C20—C19—S2	122.41 (15)
C2—C1—C6	122.44 (17)	C24—C19—S2	115.48 (14)
C6—C1—S1	122.90 (14)	C24—C19—C20	121.94 (18)
C1—S1—C7	104.77 (8)	C19—C20—H20	120.8
C1—S1—C13	103.45 (9)	C21—C20—C19	118.4 (2)
C13—S1—C7	104.88 (9)	C21—C20—H20	120.8
C1—C2—H2	120.9	C20—C21—H21	119.8
C1—C2—C3	118.25 (18)	C22—C21—C20	120.4 (2)
C3—C2—H2	120.9	C22—C21—H21	119.8
C2—C3—H3	120.0	C21—C22—H22	119.6
C4—C3—C2	120.02 (19)	C21—C22—C23	120.8 (2)
C4—C3—H3	120.0	C23—C22—H22	119.6
C3—C4—H4	119.6	C22—C23—H23	120.0
C3—C4—C5	120.71 (18)	C22—C23—C24	120.1 (2)
C5—C4—H4	119.6	C24—C23—H23	120.0
C4—C5—H5	119.9	C19—C24—C23	118.48 (18)
C4—C5—C6	120.28 (19)	C19—C24—H24	120.8
C6—C5—H5	119.9	C23—C24—H24	120.8
C1—C6—H6	120.9	C26—C25—S2	114.92 (14)
C5—C6—C1	118.26 (18)	C26—C25—C30	121.69 (18)
C5—C6—H6	120.9	C30—C25—S2	123.38 (15)
C8—C7—S1	115.83 (15)	C25—C26—H26	120.7
C8—C7—C12	121.57 (19)	C25—C26—C27	118.54 (19)
C12—C7—S1	122.60 (16)	C27—C26—H26	120.7
C7—C8—H8	120.6	C26—C27—H27	119.8
C7—C8—C9	118.85 (19)	C28—C27—C26	120.4 (2)
C9—C8—H8	120.6	C28—C27—H27	119.8
C8—C9—H9	119.9	C27—C28—H28	120.0
C10—C9—C8	120.2 (2)	C29—C28—C27	120.0 (2)
C10—C9—H9	119.9	C29—C28—H28	120.0
C9—C10—H10	120.0	C28—C29—H29	119.6
C11—C10—C9	120.1 (2)	C28—C29—C30	120.8 (2)
C11—C10—H10	120.0	C30—C29—H29	119.6
C10—C11—H11	119.4	C25—C30—H30	120.7
C10—C11—C12	121.1 (2)	C29—C30—C25	118.52 (19)
C12—C11—H11	119.4	C29—C30—H30	120.7
C7—C12—C11	118.2 (2)	C32—C31—S2	113.72 (13)
C7—C12—H12	120.9	C32—C31—C36	122.02 (17)
C11—C12—H12	120.9	C36—C31—S2	124.17 (15)
C14—C13—S1	115.34 (15)	C31—C32—H32	120.6
C14—C13—C18	121.87 (18)	C31—C32—C33	118.90 (18)
C18—C13—S1	122.79 (15)	C33—C32—H32	120.6
C13—C14—H14	120.9	C32—C33—H33	120.3
C13—C14—C15	118.18 (19)	C34—C33—C32	119.40 (19)
C15—C14—H14	120.9	C34—C33—H33	120.3
C14—C15—H15	119.7	C33—C34—H34	119.5
C16—C15—C14	120.68 (19)	C35—C34—C33	121.10 (19)
C16—C15—H15	119.7	C35—C34—H34	119.4
C15—C16—H16	119.9	C34—C35—H35	119.9
C15—C16—C17	120.16 (19)	C34—C35—C36	120.15 (18)
C17—C16—H16	119.9	C36—C35—H35	119.9
C16—C17—H17	119.8	C31—C36—C35	118.36 (18)
C18—C17—C16	120.33 (19)	C31—C36—H36	120.8
C18—C17—H17	119.8	C35—C36—H36	120.8
C13—C18—H18	120.6	Cl1—Zn1—Cl2	112.522 (19)
C17—C18—C13	118.77 (18)	Cl1—Zn1—Cl4	107.238 (18)
C17—C18—H18	120.6	Cl3—Zn1—Cl1	111.85 (2)
C25—S2—C19	106.14 (9)	Cl3—Zn1—Cl2	105.247 (19)
C31—S2—C19	100.78 (9)	Cl3—Zn1—Cl4	109.24 (2)
C31—S2—C25	106.27 (9)	Cl4—Zn1—Cl2	110.760 (19)
			
C1—S1—C7—C8	−79.65 (16)	S2—C19—C20—C21	−176.14 (16)
C1—S1—C7—C12	100.49 (18)	S2—C19—C24—C23	176.02 (15)
C1—S1—C13—C14	146.68 (15)	S2—C25—C26—C27	179.96 (15)
C1—S1—C13—C18	−32.96 (19)	S2—C25—C30—C29	179.96 (16)
C1—C2—C3—C4	0.7 (3)	S2—C31—C32—C33	−173.80 (15)
S1—C1—C2—C3	175.68 (15)	S2—C31—C36—C35	174.11 (15)
S1—C1—C6—C5	−175.80 (15)	C19—S2—C25—C26	−143.88 (14)
S1—C7—C8—C9	179.31 (15)	C19—S2—C25—C30	34.76 (19)
S1—C7—C12—C11	−179.67 (17)	C19—S2—C31—C32	79.22 (16)
S1—C13—C14—C15	−179.71 (17)	C19—S2—C31—C36	−97.33 (17)
S1—C13—C18—C17	−179.72 (16)	C19—C20—C21—C22	0.7 (3)
C2—C1—S1—C7	152.39 (15)	C20—C19—C24—C23	0.7 (3)
C2—C1—S1—C13	−97.97 (15)	C20—C21—C22—C23	0.1 (3)
C2—C1—C6—C5	1.9 (3)	C21—C22—C23—C24	−0.6 (3)
C2—C3—C4—C5	1.1 (3)	C22—C23—C24—C19	0.2 (3)
C3—C4—C5—C6	−1.4 (3)	C24—C19—C20—C21	−1.2 (3)
C4—C5—C6—C1	−0.1 (3)	C25—S2—C19—C20	−78.26 (19)
C6—C1—S1—C7	−29.71 (18)	C25—S2—C19—C24	106.50 (15)
C6—C1—S1—C13	79.93 (18)	C25—S2—C31—C32	−170.27 (14)
C6—C1—C2—C3	−2.2 (3)	C25—S2—C31—C36	13.18 (19)
C7—S1—C13—C14	−103.76 (16)	C25—C26—C27—C28	−0.2 (3)
C7—S1—C13—C18	76.59 (19)	C26—C25—C30—C29	−1.5 (3)
C7—C8—C9—C10	0.7 (3)	C26—C27—C28—C29	−0.7 (3)
C8—C7—C12—C11	0.5 (3)	C27—C28—C29—C30	0.5 (3)
C8—C9—C10—C11	−0.3 (3)	C28—C29—C30—C25	0.6 (3)
C9—C10—C11—C12	0.0 (3)	C30—C25—C26—C27	1.3 (3)
C10—C11—C12—C7	0.0 (3)	C31—S2—C19—C20	32.35 (19)
C12—C7—C8—C9	−0.8 (3)	C31—S2—C19—C24	−142.89 (15)
C13—S1—C7—C8	171.75 (15)	C31—S2—C25—C26	109.43 (15)
C13—S1—C7—C12	−8.11 (19)	C31—S2—C25—C30	−71.94 (18)
C13—C14—C15—C16	−0.4 (3)	C31—C32—C33—C34	−1.3 (3)
C14—C13—C18—C17	0.7 (3)	C32—C31—C36—C35	−2.2 (3)
C14—C15—C16—C17	0.3 (4)	C32—C33—C34—C35	−0.8 (3)
C15—C16—C17—C18	0.3 (3)	C33—C34—C35—C36	1.4 (3)
C16—C17—C18—C13	−0.8 (3)	C34—C35—C36—C31	0.0 (3)
C18—C13—C14—C15	−0.1 (3)	C36—C31—C32—C33	2.8 (3)

### Bis(triphenylsulfonium) tetrachloridozinc(II) (I) Hydrogen-bond geometry (Å, º)

**Table:**

*D*—H···*A*	*D*—H	H···*A*	*D*···*A*	*D*—H···*A*
C3—H3···Cl3^i^	0.95	2.68	3.5210 (19)	148
C8—H8···Cl2	0.95	2.66	3.552 (2)	158
C14—H14···Cl1	0.95	2.75	3.610 (2)	151
C21—H21···Cl1^ii^	0.95	2.65	3.554 (2)	159
C24—H24···Cl4	0.95	2.74	3.5792 (19)	147
C26—H26···Cl2	0.95	2.66	3.509 (2)	149
C33—H33···Cl3^i^	0.95	2.76	3.695 (2)	167
C35—H35···Cl1^iii^	0.95	2.81	3.592 (2)	140

Symmetry codes: (i) −*x*+1, −*y*+1, −*z*+1; (ii) *x*−1/2, −*y*+3/2, *z*+1/2; (iii) *x*+1/2, −*y*+3/2, *z*+1/2.

### Bis(triphenylsulfonium) tetrachloridocadmium(II) (II) Crystal data

**Table:**

(C_18_H_15_S)_2_[CdCl_4_]	*F*(000) = 1576
*M**_r_* = 780.92	*D*_x_ = 1.501 Mg m^−^^3^
Monoclinic, *P*2_1_/*n*	Cu *K*α radiation, λ = 1.54184 Å
*a* = 9.2696 (1) Å	Cell parameters from 13508 reflections
*b* = 17.6682 (2) Å	θ = 3.3–69.5°
*c* = 21.4072 (2) Å	µ = 9.22 mm^−^^1^
β = 99.801 (1)°	*T* = 100 K
*V* = 3454.84 (6) Å^3^	Irregular, clear colourless
*Z* = 4	0.19 × 0.13 × 0.09 mm

### Bis(triphenylsulfonium) tetrachloridocadmium(II) (II) Data collection

**Table:**

XtaLAB Synergy, Single source at home/near, HyPix3000 diffractometer	5793 reflections with *I* > 2σ(*I*)
Detector resolution: 10.0000 pixels mm^-1^	*R*_int_ = 0.055
ω scans	θ_max_ = 69.9°, θ_min_ = 3.3°
Absorption correction: multi-scan (CrysAlisPro; Rigaku OD, 2023)	*h* = −11→11
*T*_min_ = 0.488, *T*_max_ = 1.000	*k* = −21→21
20400 measured reflections	*l* = −21→25
6456 independent reflections	

### Bis(triphenylsulfonium) tetrachloridocadmium(II) (II) Refinement

**Table:**

Refinement on *F*^2^	0 restraints
Least-squares matrix: full	Hydrogen site location: inferred from neighbouring sites
*R*[*F*^2^ > 2σ(*F*^2^)] = 0.026	H-atom parameters constrained
*wR*(*F*^2^) = 0.066	*w* = 1/[σ^2^(*F*_o_^2^) + (0.0269*P*)^2^ + 1.3464*P*] where *P* = (*F*_o_^2^ + 2*F*_c_^2^)/3
*S* = 1.05	(Δ/σ)_max_ = 0.002
6456 reflections	Δρ_max_ = 0.63 e Å^−^^3^
388 parameters	Δρ_min_ = −0.49 e Å^−^^3^

### Bis(triphenylsulfonium) tetrachloridocadmium(II) (II) Special details

**Table:**

Geometry. All esds (except the esd in the dihedral angle between two l.s. planes) are estimated using the full covariance matrix. The cell esds are taken into account individually in the estimation of esds in distances, angles and torsion angles; correlations between esds in cell parameters are only used when they are defined by crystal symmetry. An approximate (isotropic) treatment of cell esds is used for estimating esds involving l.s. planes.

### Bis(triphenylsulfonium) tetrachloridocadmium(II) (II) Fractional atomic coordinates and isotropic or equivalent isotropic displacement parameters (Å^2^)

**Table:**

	*x*	*y*	*z*	*U*_iso_*/*U*_eq_	
C1	0.0953 (3)	0.46409 (14)	0.72725 (11)	0.0276 (5)	
S1	0.24934 (6)	0.48478 (3)	0.68954 (2)	0.02528 (12)	
C2	0.0226 (3)	0.39737 (17)	0.70845 (12)	0.0378 (6)	
H2	0.054940	0.365675	0.677752	0.045*	
C3	−0.0987 (3)	0.37742 (18)	0.73523 (13)	0.0407 (6)	
H3	−0.149570	0.331665	0.722960	0.049*	
C4	−0.1450 (3)	0.42379 (17)	0.77937 (13)	0.0401 (7)	
H4	−0.227748	0.410058	0.797649	0.048*	
C5	−0.0724 (4)	0.48939 (18)	0.79694 (16)	0.0530 (9)	
H5	−0.105972	0.521259	0.827213	0.064*	
C6	0.0498 (4)	0.51075 (17)	0.77156 (13)	0.0450 (7)	
H6	0.100554	0.556347	0.784451	0.054*	
C7	0.1726 (3)	0.52561 (14)	0.61506 (10)	0.0254 (5)	
C8	0.0427 (3)	0.56613 (15)	0.60619 (12)	0.0313 (5)	
H8	−0.014015	0.570429	0.639026	0.038*	
C9	−0.0018 (3)	0.60040 (15)	0.54733 (12)	0.0346 (6)	
H9	−0.090013	0.628812	0.539664	0.041*	
C10	0.0818 (3)	0.59315 (15)	0.50038 (12)	0.0351 (6)	
H10	0.051726	0.617640	0.460805	0.042*	
C11	0.2082 (3)	0.55103 (16)	0.50987 (12)	0.0344 (6)	
H11	0.263113	0.545746	0.476496	0.041*	
C12	0.2564 (3)	0.51612 (15)	0.56770 (11)	0.0286 (5)	
H12	0.343587	0.486803	0.574643	0.034*	
C13	0.3395 (3)	0.56290 (15)	0.73269 (11)	0.0288 (5)	
C14	0.2880 (3)	0.63655 (16)	0.72499 (12)	0.0364 (6)	
H14	0.200994	0.647640	0.696081	0.044*	
C15	0.3652 (3)	0.69340 (16)	0.75994 (14)	0.0408 (7)	
H15	0.330866	0.744102	0.755397	0.049*	
C16	0.4922 (3)	0.67695 (17)	0.80156 (13)	0.0420 (7)	
H16	0.544911	0.716556	0.825329	0.050*	
C17	0.5430 (3)	0.60427 (19)	0.80894 (15)	0.0491 (8)	
H17	0.630512	0.593750	0.837747	0.059*	
C18	0.4671 (3)	0.54570 (17)	0.77447 (13)	0.0397 (6)	
H18	0.501758	0.495080	0.779359	0.048*	
S2	0.54251 (6)	0.77489 (3)	0.49517 (2)	0.02358 (12)	
C19	0.6295 (3)	0.72077 (14)	0.56160 (10)	0.0253 (5)	
C20	0.7241 (3)	0.75133 (15)	0.61263 (11)	0.0300 (5)	
H20	0.755286	0.802509	0.612185	0.036*	
C21	0.7717 (3)	0.70487 (16)	0.66432 (11)	0.0344 (6)	
H21	0.836181	0.724422	0.699929	0.041*	
C22	0.7261 (3)	0.63053 (17)	0.66441 (12)	0.0361 (6)	
H22	0.756739	0.599831	0.700676	0.043*	
C23	0.6361 (3)	0.60025 (16)	0.61203 (13)	0.0371 (6)	
H23	0.607569	0.548609	0.612030	0.044*	
C24	0.5877 (3)	0.64525 (15)	0.55971 (11)	0.0309 (5)	
H24	0.527256	0.624881	0.523306	0.037*	
C25	0.6324 (3)	0.86513 (14)	0.50027 (11)	0.0288 (5)	
C26	0.6108 (3)	0.91985 (16)	0.54382 (12)	0.0391 (6)	
H26	0.548072	0.910846	0.573824	0.047*	
C27	0.6839 (4)	0.98893 (17)	0.54242 (14)	0.0473 (7)	
H27	0.670464	1.027677	0.571653	0.057*	
C28	0.7753 (4)	1.00122 (17)	0.49902 (15)	0.0463 (7)	
H28	0.825888	1.047982	0.499060	0.056*	
C29	0.7938 (3)	0.94590 (18)	0.45535 (15)	0.0445 (7)	
H29	0.855639	0.955080	0.425033	0.053*	
C30	0.7222 (3)	0.87703 (16)	0.45580 (13)	0.0343 (6)	
H30	0.734521	0.838686	0.426058	0.041*	
C31	0.3665 (3)	0.79086 (14)	0.51693 (11)	0.0271 (5)	
C32	0.2482 (3)	0.78776 (14)	0.46805 (12)	0.0299 (5)	
H32	0.262164	0.780592	0.425510	0.036*	
C33	0.1084 (3)	0.79537 (16)	0.48249 (15)	0.0389 (6)	
H33	0.025450	0.793564	0.449647	0.047*	
C34	0.0902 (3)	0.80549 (17)	0.54425 (17)	0.0454 (7)	
H34	−0.005668	0.810894	0.553847	0.055*	
C35	0.2102 (4)	0.8079 (2)	0.59286 (15)	0.0510 (8)	
H35	0.196177	0.815147	0.635394	0.061*	
C36	0.3492 (3)	0.79992 (18)	0.57954 (13)	0.0413 (7)	
H36	0.431866	0.800567	0.612574	0.050*	
Cd1	0.03901 (2)	0.80558 (2)	0.83501 (2)	0.02284 (6)	
Cl1	−0.10045 (7)	0.89355 (4)	0.88926 (3)	0.03605 (14)	
Cl2	0.04029 (7)	0.84236 (4)	0.72454 (3)	0.03335 (14)	
Cl3	0.28460 (6)	0.80632 (3)	0.90087 (3)	0.02929 (13)	
Cl4	−0.07390 (7)	0.67949 (4)	0.83420 (3)	0.03362 (14)	

### Bis(triphenylsulfonium) tetrachloridocadmium(II) (II) Atomic displacement parameters (Å^2^)

**Table:**

	*U*^11^	*U*^22^	*U*^33^	*U*^12^	*U*^13^	*U*^23^
C1	0.0306 (12)	0.0277 (13)	0.0242 (11)	0.0055 (10)	0.0034 (9)	0.0039 (10)
S1	0.0274 (3)	0.0239 (3)	0.0233 (2)	0.0032 (2)	0.0011 (2)	0.0011 (2)
C2	0.0349 (14)	0.0441 (17)	0.0350 (13)	−0.0019 (12)	0.0080 (11)	−0.0101 (12)
C3	0.0347 (14)	0.0457 (17)	0.0424 (14)	−0.0050 (13)	0.0083 (12)	−0.0041 (13)
C4	0.0438 (16)	0.0399 (16)	0.0408 (14)	0.0078 (13)	0.0188 (12)	0.0121 (13)
C5	0.079 (2)	0.0359 (17)	0.0556 (18)	0.0047 (16)	0.0438 (18)	0.0011 (15)
C6	0.070 (2)	0.0292 (15)	0.0425 (15)	−0.0062 (14)	0.0279 (15)	−0.0051 (12)
C7	0.0295 (12)	0.0228 (12)	0.0225 (10)	0.0000 (10)	0.0003 (9)	0.0007 (9)
C8	0.0321 (13)	0.0308 (14)	0.0298 (12)	0.0050 (11)	0.0025 (10)	0.0004 (11)
C9	0.0358 (14)	0.0262 (14)	0.0380 (13)	0.0036 (11)	−0.0043 (11)	0.0028 (11)
C10	0.0459 (15)	0.0277 (14)	0.0283 (12)	−0.0111 (12)	−0.0036 (11)	0.0078 (11)
C11	0.0380 (14)	0.0384 (15)	0.0282 (12)	−0.0104 (12)	0.0093 (11)	0.0046 (11)
C12	0.0242 (12)	0.0290 (13)	0.0327 (12)	−0.0030 (10)	0.0050 (10)	0.0010 (10)
C13	0.0314 (13)	0.0266 (13)	0.0266 (11)	−0.0005 (10)	−0.0001 (10)	−0.0019 (10)
C14	0.0380 (14)	0.0322 (15)	0.0358 (13)	0.0023 (12)	−0.0031 (11)	−0.0027 (11)
C15	0.0489 (17)	0.0281 (15)	0.0419 (15)	0.0018 (12)	−0.0019 (13)	−0.0048 (12)
C16	0.0456 (16)	0.0349 (16)	0.0402 (14)	−0.0034 (13)	−0.0078 (13)	−0.0079 (12)
C17	0.0426 (16)	0.0434 (18)	0.0524 (17)	0.0026 (14)	−0.0175 (14)	−0.0042 (14)
C18	0.0372 (14)	0.0317 (15)	0.0447 (15)	0.0049 (12)	−0.0087 (12)	−0.0017 (12)
S2	0.0241 (3)	0.0237 (3)	0.0228 (2)	−0.0017 (2)	0.0038 (2)	−0.0005 (2)
C19	0.0257 (12)	0.0266 (12)	0.0236 (11)	0.0000 (10)	0.0040 (9)	0.0006 (10)
C20	0.0306 (13)	0.0283 (13)	0.0304 (12)	−0.0024 (11)	0.0034 (10)	−0.0061 (10)
C21	0.0317 (13)	0.0435 (16)	0.0254 (12)	−0.0028 (12)	−0.0023 (10)	−0.0031 (11)
C22	0.0344 (14)	0.0423 (16)	0.0308 (12)	0.0034 (12)	0.0032 (10)	0.0098 (12)
C23	0.0376 (14)	0.0287 (14)	0.0440 (14)	−0.0047 (11)	0.0042 (12)	0.0086 (12)
C24	0.0297 (13)	0.0298 (14)	0.0308 (12)	−0.0062 (11)	−0.0012 (10)	−0.0023 (10)
C25	0.0306 (13)	0.0227 (12)	0.0311 (12)	−0.0020 (10)	−0.0005 (10)	0.0030 (10)
C26	0.0544 (18)	0.0306 (15)	0.0325 (13)	−0.0022 (13)	0.0077 (12)	−0.0036 (11)
C27	0.069 (2)	0.0284 (15)	0.0423 (15)	−0.0057 (14)	0.0026 (15)	−0.0063 (13)
C28	0.0524 (18)	0.0277 (15)	0.0559 (17)	−0.0111 (13)	0.0009 (14)	0.0025 (13)
C29	0.0431 (16)	0.0385 (17)	0.0539 (17)	−0.0095 (13)	0.0141 (14)	0.0034 (14)
C30	0.0334 (13)	0.0290 (14)	0.0415 (14)	−0.0022 (11)	0.0093 (11)	−0.0009 (11)
C31	0.0272 (12)	0.0250 (12)	0.0311 (12)	0.0015 (10)	0.0101 (10)	0.0032 (10)
C32	0.0281 (13)	0.0241 (13)	0.0373 (13)	−0.0026 (10)	0.0052 (10)	−0.0035 (11)
C33	0.0266 (13)	0.0299 (15)	0.0596 (18)	−0.0035 (11)	0.0052 (12)	−0.0014 (13)
C34	0.0341 (15)	0.0378 (17)	0.071 (2)	0.0047 (13)	0.0277 (15)	0.0130 (15)
C35	0.057 (2)	0.059 (2)	0.0445 (16)	0.0181 (16)	0.0299 (15)	0.0154 (15)
C36	0.0399 (16)	0.0548 (19)	0.0319 (13)	0.0118 (14)	0.0132 (12)	0.0086 (13)
Cd1	0.02514 (9)	0.02262 (9)	0.02076 (8)	−0.00049 (6)	0.00392 (6)	−0.00405 (6)
Cl1	0.0372 (3)	0.0373 (3)	0.0345 (3)	0.0079 (3)	0.0083 (2)	−0.0117 (3)
Cl2	0.0461 (3)	0.0329 (3)	0.0222 (2)	−0.0039 (3)	0.0093 (2)	−0.0037 (2)
Cl3	0.0252 (3)	0.0286 (3)	0.0322 (3)	−0.0027 (2)	−0.0003 (2)	−0.0001 (2)
Cl4	0.0412 (3)	0.0280 (3)	0.0303 (3)	−0.0100 (3)	0.0022 (2)	−0.0059 (2)

### Bis(triphenylsulfonium) tetrachloridocadmium(II) (II) Geometric parameters (Å, º)

**Table:**

C1—S1	1.794 (3)	S2—C31	1.794 (2)
C1—C2	1.383 (4)	C19—C20	1.388 (3)
C1—C6	1.376 (4)	C19—C24	1.388 (4)
S1—C7	1.784 (2)	C20—H20	0.9500
S1—C13	1.787 (3)	C20—C21	1.388 (4)
C2—H2	0.9500	C21—H21	0.9500
C2—C3	1.392 (4)	C21—C22	1.380 (4)
C3—H3	0.9500	C22—H22	0.9500
C3—C4	1.373 (4)	C22—C23	1.386 (4)
C4—H4	0.9500	C23—H23	0.9500
C4—C5	1.361 (5)	C23—C24	1.384 (4)
C5—H5	0.9500	C24—H24	0.9500
C5—C6	1.389 (4)	C25—C26	1.381 (4)
C6—H6	0.9500	C25—C30	1.383 (4)
C7—C8	1.386 (3)	C26—H26	0.9500
C7—C12	1.388 (3)	C26—C27	1.399 (4)
C8—H8	0.9500	C27—H27	0.9500
C8—C9	1.396 (4)	C27—C28	1.377 (5)
C9—H9	0.9500	C28—H28	0.9500
C9—C10	1.376 (4)	C28—C29	1.383 (4)
C10—H10	0.9500	C29—H29	0.9500
C10—C11	1.373 (4)	C29—C30	1.387 (4)
C11—H11	0.9500	C30—H30	0.9500
C11—C12	1.387 (4)	C31—C32	1.382 (4)
C12—H12	0.9500	C31—C36	1.386 (4)
C13—C14	1.386 (4)	C32—H32	0.9500
C13—C18	1.390 (4)	C32—C33	1.389 (4)
C14—H14	0.9500	C33—H33	0.9500
C14—C15	1.378 (4)	C33—C34	1.373 (4)
C15—H15	0.9500	C34—H34	0.9500
C15—C16	1.381 (4)	C34—C35	1.389 (5)
C16—H16	0.9500	C35—H35	0.9500
C16—C17	1.367 (4)	C35—C36	1.374 (4)
C17—H17	0.9500	C36—H36	0.9500
C17—C18	1.390 (4)	Cd1—Cl1	2.4386 (6)
C18—H18	0.9500	Cd1—Cl2	2.4545 (6)
S2—C19	1.788 (2)	Cd1—Cl3	2.4653 (6)
S2—C25	1.794 (3)	Cd1—Cl4	2.4602 (6)
			
C2—C1—S1	115.38 (19)	C20—C19—S2	123.8 (2)
C6—C1—S1	123.3 (2)	C24—C19—S2	113.91 (17)
C6—C1—C2	121.3 (3)	C24—C19—C20	122.2 (2)
C7—S1—C1	104.94 (11)	C19—C20—H20	121.0
C7—S1—C13	103.29 (12)	C21—C20—C19	118.0 (2)
C13—S1—C1	105.24 (12)	C21—C20—H20	121.0
C1—C2—H2	120.5	C20—C21—H21	119.7
C1—C2—C3	118.9 (3)	C22—C21—C20	120.5 (2)
C3—C2—H2	120.5	C22—C21—H21	119.7
C2—C3—H3	120.0	C21—C22—H22	119.7
C4—C3—C2	120.1 (3)	C21—C22—C23	120.6 (2)
C4—C3—H3	120.0	C23—C22—H22	119.7
C3—C4—H4	120.0	C22—C23—H23	120.0
C5—C4—C3	120.0 (3)	C24—C23—C22	120.0 (3)
C5—C4—H4	120.0	C24—C23—H23	120.0
C4—C5—H5	119.3	C19—C24—H24	120.7
C4—C5—C6	121.4 (3)	C23—C24—C19	118.6 (2)
C6—C5—H5	119.3	C23—C24—H24	120.7
C1—C6—C5	118.2 (3)	C26—C25—S2	122.9 (2)
C1—C6—H6	120.9	C26—C25—C30	122.1 (3)
C5—C6—H6	120.9	C30—C25—S2	114.9 (2)
C8—C7—S1	122.43 (18)	C25—C26—H26	121.0
C8—C7—C12	122.9 (2)	C25—C26—C27	118.0 (3)
C12—C7—S1	114.60 (18)	C27—C26—H26	121.0
C7—C8—H8	121.2	C26—C27—H27	119.7
C7—C8—C9	117.7 (2)	C28—C27—C26	120.5 (3)
C9—C8—H8	121.2	C28—C27—H27	119.7
C8—C9—H9	119.9	C27—C28—H28	119.8
C10—C9—C8	120.2 (3)	C27—C28—C29	120.4 (3)
C10—C9—H9	119.9	C29—C28—H28	119.8
C9—C10—H10	119.5	C28—C29—H29	120.0
C11—C10—C9	121.0 (2)	C28—C29—C30	120.0 (3)
C11—C10—H10	119.5	C30—C29—H29	120.0
C10—C11—H11	119.7	C25—C30—C29	118.9 (3)
C10—C11—C12	120.7 (2)	C25—C30—H30	120.6
C12—C11—H11	119.7	C29—C30—H30	120.6
C7—C12—H12	121.2	C32—C31—S2	115.91 (19)
C11—C12—C7	117.5 (2)	C32—C31—C36	122.0 (2)
C11—C12—H12	121.2	C36—C31—S2	121.9 (2)
C14—C13—S1	122.84 (19)	C31—C32—H32	120.7
C14—C13—C18	121.3 (2)	C31—C32—C33	118.6 (2)
C18—C13—S1	115.8 (2)	C33—C32—H32	120.7
C13—C14—H14	120.5	C32—C33—H33	120.0
C15—C14—C13	118.9 (2)	C34—C33—C32	120.0 (3)
C15—C14—H14	120.5	C34—C33—H33	120.0
C14—C15—H15	119.9	C33—C34—H34	119.6
C14—C15—C16	120.2 (3)	C33—C34—C35	120.8 (3)
C16—C15—H15	119.9	C35—C34—H34	119.6
C15—C16—H16	119.6	C34—C35—H35	119.9
C17—C16—C15	120.7 (3)	C36—C35—C34	120.1 (3)
C17—C16—H16	119.6	C36—C35—H35	119.9
C16—C17—H17	119.9	C31—C36—H36	120.7
C16—C17—C18	120.3 (3)	C35—C36—C31	118.7 (3)
C18—C17—H17	119.9	C35—C36—H36	120.7
C13—C18—C17	118.5 (3)	Cl1—Cd1—Cl2	112.33 (2)
C13—C18—H18	120.8	Cl1—Cd1—Cl3	103.71 (2)
C17—C18—H18	120.8	Cl1—Cd1—Cl4	108.73 (2)
C19—S2—C25	106.56 (11)	Cl2—Cd1—Cl3	113.21 (2)
C19—S2—C31	100.63 (11)	Cl2—Cd1—Cl4	107.74 (2)
C31—S2—C25	106.16 (12)	Cl4—Cd1—Cl3	111.05 (2)
			
C1—S1—C7—C8	29.3 (2)	S2—C19—C20—C21	173.1 (2)
C1—S1—C7—C12	−153.19 (19)	S2—C19—C24—C23	−173.0 (2)
C1—S1—C13—C14	−77.0 (2)	S2—C25—C26—C27	−178.4 (2)
C1—S1—C13—C18	103.8 (2)	S2—C25—C30—C29	178.7 (2)
C1—C2—C3—C4	0.3 (4)	S2—C31—C32—C33	176.4 (2)
S1—C1—C2—C3	−179.6 (2)	S2—C31—C36—C35	−176.6 (2)
S1—C1—C6—C5	179.1 (2)	C19—S2—C25—C26	−74.1 (2)
S1—C7—C8—C9	175.3 (2)	C19—S2—C25—C30	107.8 (2)
S1—C7—C12—C11	−175.59 (19)	C19—S2—C31—C32	−141.6 (2)
S1—C13—C14—C15	−179.5 (2)	C19—S2—C31—C36	33.6 (3)
S1—C13—C18—C17	179.4 (2)	C19—C20—C21—C22	0.2 (4)
C2—C1—S1—C7	81.7 (2)	C20—C19—C24—C23	3.5 (4)
C2—C1—S1—C13	−169.7 (2)	C20—C21—C22—C23	2.2 (4)
C2—C1—C6—C5	−0.4 (4)	C21—C22—C23—C24	−1.8 (4)
C2—C3—C4—C5	0.1 (5)	C22—C23—C24—C19	−1.0 (4)
C3—C4—C5—C6	−0.6 (5)	C24—C19—C20—C21	−3.2 (4)
C4—C5—C6—C1	0.8 (5)	C25—S2—C19—C20	12.6 (2)
C6—C1—S1—C7	−97.8 (2)	C25—S2—C19—C24	−170.83 (19)
C6—C1—S1—C13	10.8 (3)	C25—S2—C31—C32	107.5 (2)
C6—C1—C2—C3	−0.1 (4)	C25—S2—C31—C36	−77.3 (2)
C7—S1—C13—C14	32.8 (3)	C25—C26—C27—C28	−0.4 (5)
C7—S1—C13—C18	−146.4 (2)	C26—C25—C30—C29	0.6 (4)
C7—C8—C9—C10	0.3 (4)	C26—C27—C28—C29	1.2 (5)
C8—C7—C12—C11	1.9 (4)	C27—C28—C29—C30	−1.1 (5)
C8—C9—C10—C11	1.4 (4)	C28—C29—C30—C25	0.2 (4)
C9—C10—C11—C12	−1.5 (4)	C30—C25—C26—C27	−0.5 (4)
C10—C11—C12—C7	−0.1 (4)	C31—S2—C19—C20	−97.9 (2)
C12—C7—C8—C9	−2.0 (4)	C31—S2—C19—C24	78.6 (2)
C13—S1—C7—C8	−80.7 (2)	C31—S2—C25—C26	32.5 (3)
C13—S1—C7—C12	96.8 (2)	C31—S2—C25—C30	−145.5 (2)
C13—C14—C15—C16	0.5 (4)	C31—C32—C33—C34	−0.2 (4)
C14—C13—C18—C17	0.2 (4)	C32—C31—C36—C35	−1.7 (4)
C14—C15—C16—C17	−0.3 (5)	C32—C33—C34—C35	−0.3 (4)
C15—C16—C17—C18	0.0 (5)	C33—C34—C35—C36	−0.2 (5)
C16—C17—C18—C13	0.0 (5)	C34—C35—C36—C31	1.2 (5)
C18—C13—C14—C15	−0.4 (4)	C36—C31—C32—C33	1.2 (4)

### Bis(triphenylsulfonium) tetrachloridocadmium(II) (II) Hydrogen-bond geometry (Å, º)

**Table:**

*D*—H···*A*	*D*—H	H···*A*	*D*···*A*	*D*—H···*A*
C2—H2···Cl3^i^	0.95	2.65	3.561 (3)	162
C5—H5···Cl4	0.95	2.81	3.453 (3)	126
C11—H11···Cl1^ii^	0.95	2.65	3.512 (3)	150
C18—H18···Cl2^i^	0.95	2.73	3.593 (3)	152
C21—H21···Cl2^iii^	0.95	2.80	3.559 (3)	137
C23—H23···Cl1^i^	0.95	2.74	3.667 (3)	165
C30—H30···Cl3^ii^	0.95	2.67	3.528 (3)	150
C32—H32···Cl4^ii^	0.95	2.76	3.588 (3)	146
C35—H35···Cl2	0.95	2.63	3.508 (3)	155

Symmetry codes: (i) −*x*+1/2, *y*−1/2, −*z*+3/2; (ii) *x*+1/2, −*y*+3/2, *z*−1/2; (iii) *x*+1, *y*, *z*.

### Bis(triphenylsulfonium) tetrachloridomercury(II) methanol monosolvate (III) Crystal data

**Table:**

(C_18_H_15_S)_2_[HgCl_4_]·CH_4_O	*F*(000) = 1776
*M**_r_* = 901.15	*D*_x_ = 1.659 Mg m^−^^3^
Monoclinic, *P*2_1_/*n*	Cu *K*α radiation, λ = 1.54184 Å
*a* = 9.43577 (14) Å	Cell parameters from 16563 reflections
*b* = 18.0709 (3) Å	θ = 3.2–69.7°
*c* = 21.2467 (3) Å	µ = 11.68 mm^−^^1^
β = 95.1778 (14)°	*T* = 100 K
*V* = 3608.05 (9) Å^3^	Irregular, clear colourless
*Z* = 4	0.11 × 0.06 × 0.05 mm

### Bis(triphenylsulfonium) tetrachloridomercury(II) methanol monosolvate (III) Data collection

**Table:**

XtaLAB Synergy, Single source at home/near, HyPix3000 diffractometer	6142 reflections with *I* > 2σ(*I*)
Detector resolution: 10.0000 pixels mm^-1^	*R*_int_ = 0.054
ω scans	θ_max_ = 70.0°, θ_min_ = 3.2°
Absorption correction: multi-scan (CrysAlisPro; Rigaku OD, 2023)	*h* = −11→11
*T*_min_ = 0.644, *T*_max_ = 1.000	*k* = −17→21
22094 measured reflections	*l* = −25→25
6743 independent reflections	

### Bis(triphenylsulfonium) tetrachloridomercury(II) methanol monosolvate (III) Refinement

**Table:**

Refinement on *F*^2^	0 restraints
Least-squares matrix: full	Hydrogen site location: inferred from neighbouring sites
*R*[*F*^2^ > 2σ(*F*^2^)] = 0.031	H-atom parameters constrained
*wR*(*F*^2^) = 0.080	*w* = 1/[σ^2^(*F*_o_^2^) + (0.0299*P*)^2^ + 13.4612*P*] where *P* = (*F*_o_^2^ + 2*F*_c_^2^)/3
*S* = 1.07	(Δ/σ)_max_ = 0.002
6743 reflections	Δρ_max_ = 2.45 e Å^−^^3^
408 parameters	Δρ_min_ = −2.10 e Å^−^^3^

### Bis(triphenylsulfonium) tetrachloridomercury(II) methanol monosolvate (III) Special details

**Table:**

Geometry. All esds (except the esd in the dihedral angle between two l.s. planes) are estimated using the full covariance matrix. The cell esds are taken into account individually in the estimation of esds in distances, angles and torsion angles; correlations between esds in cell parameters are only used when they are defined by crystal symmetry. An approximate (isotropic) treatment of cell esds is used for estimating esds involving l.s. planes.

### Bis(triphenylsulfonium) tetrachloridomercury(II) methanol monosolvate (III) Fractional atomic coordinates and isotropic or equivalent isotropic displacement parameters (Å^2^)

**Table:**

	*x*	*y*	*z*	*U*_iso_*/*U*_eq_	
Cl1	0.59944 (13)	0.41086 (6)	0.70016 (5)	0.0333 (2)	
Hg1	0.54319 (2)	0.35810 (2)	0.80553 (2)	0.02241 (7)	
Cl2	0.37497 (11)	0.25678 (6)	0.77766 (5)	0.0293 (2)	
Cl3	0.76456 (11)	0.32714 (6)	0.87198 (5)	0.0282 (2)	
Cl4	0.44023 (12)	0.45546 (7)	0.86876 (6)	0.0366 (3)	
S2	0.78402 (10)	0.52803 (5)	0.82731 (5)	0.0201 (2)	
C20	0.9390 (4)	0.5172 (2)	0.78599 (19)	0.0211 (8)	
C21	0.9934 (5)	0.4454 (2)	0.7864 (2)	0.0272 (9)	
H21	0.946053	0.406119	0.805593	0.033*	
C22	1.1182 (5)	0.4324 (3)	0.7581 (2)	0.0340 (11)	
H22	1.157445	0.384013	0.758005	0.041*	
C23	1.1851 (5)	0.4902 (3)	0.7302 (2)	0.0309 (10)	
H23	1.271514	0.481399	0.711697	0.037*	
C24	1.1277 (5)	0.5606 (3)	0.7290 (2)	0.0290 (10)	
H24	1.173856	0.599328	0.708548	0.035*	
C25	1.0038 (5)	0.5755 (2)	0.7570 (2)	0.0237 (9)	
H25	0.964611	0.623926	0.756497	0.028*	
C26	0.6957 (4)	0.6094 (2)	0.7969 (2)	0.0214 (8)	
C27	0.7468 (5)	0.6803 (3)	0.8110 (2)	0.0297 (10)	
H27	0.833578	0.687344	0.836611	0.036*	
C28	0.6686 (5)	0.7404 (3)	0.7869 (2)	0.0344 (11)	
H28	0.701249	0.789254	0.796272	0.041*	
C29	0.5431 (5)	0.7295 (3)	0.7492 (2)	0.0318 (10)	
H29	0.490540	0.770944	0.732394	0.038*	
C30	0.4938 (5)	0.6590 (3)	0.7358 (2)	0.0329 (11)	
H30	0.407356	0.652281	0.709896	0.039*	
C31	0.5691 (4)	0.5979 (3)	0.7598 (2)	0.0249 (9)	
H31	0.534816	0.549215	0.750944	0.030*	
C32	0.8516 (5)	0.5553 (2)	0.9050 (2)	0.0239 (9)	
C33	0.9851 (5)	0.5856 (3)	0.9187 (2)	0.0316 (10)	
H33	1.047862	0.591326	0.886503	0.038*	
C34	1.0261 (5)	0.6075 (3)	0.9800 (2)	0.0354 (11)	
H34	1.117493	0.628649	0.990115	0.042*	
C35	0.9349 (5)	0.5989 (3)	1.0265 (2)	0.0347 (11)	
H35	0.963887	0.614196	1.068491	0.042*	
C36	0.8016 (6)	0.5680 (3)	1.0127 (2)	0.0411 (13)	
H36	0.739470	0.562244	1.045180	0.049*	
C37	0.7582 (5)	0.5455 (3)	0.9513 (2)	0.0340 (11)	
H37	0.667161	0.523937	0.941264	0.041*	
S1	0.98108 (10)	0.26645 (6)	0.44256 (5)	0.0219 (2)	
C2	0.8066 (4)	0.2824 (2)	0.4665 (2)	0.0230 (9)	
C3	0.6955 (5)	0.2756 (2)	0.4193 (2)	0.0255 (9)	
H3	0.713154	0.261399	0.377722	0.031*	
C4	0.5587 (5)	0.2900 (3)	0.4343 (2)	0.0331 (11)	
H4	0.481568	0.286110	0.402476	0.040*	
C5	0.5328 (5)	0.3097 (3)	0.4947 (2)	0.0348 (11)	
H5	0.438249	0.319746	0.504337	0.042*	
C6	0.6444 (5)	0.3151 (3)	0.5417 (2)	0.0345 (11)	
H6	0.625593	0.328103	0.583410	0.041*	
C7	0.7827 (5)	0.3016 (3)	0.5283 (2)	0.0310 (10)	
H7	0.859459	0.305301	0.560330	0.037*	
C8	1.0795 (4)	0.2404 (2)	0.5151 (2)	0.0235 (9)	
C9	1.0712 (5)	0.1663 (3)	0.5299 (2)	0.0312 (10)	
H9	1.022355	0.132622	0.501231	0.037*	
C10	1.1353 (6)	0.1421 (3)	0.5873 (3)	0.0406 (13)	
H10	1.129932	0.091453	0.598692	0.049*	
C11	1.2080 (5)	0.1922 (3)	0.6283 (2)	0.0334 (11)	
H11	1.250484	0.175755	0.668052	0.040*	
C12	1.2185 (5)	0.2659 (3)	0.6115 (2)	0.0318 (10)	
H12	1.270571	0.299375	0.639192	0.038*	
C13	1.1533 (5)	0.2910 (3)	0.5541 (2)	0.0268 (9)	
H13	1.159333	0.341526	0.542120	0.032*	
C14	1.0444 (5)	0.3571 (2)	0.4246 (2)	0.0236 (9)	
C15	0.9691 (5)	0.4200 (3)	0.4365 (2)	0.0319 (10)	
H15	0.883316	0.416831	0.456542	0.038*	
C16	1.0206 (6)	0.4879 (3)	0.4188 (2)	0.0365 (11)	
H16	0.968433	0.531666	0.425789	0.044*	
C17	1.1475 (6)	0.4926 (3)	0.3909 (2)	0.0341 (11)	
H17	1.183336	0.539392	0.379630	0.041*	
C18	1.2218 (5)	0.4284 (3)	0.3795 (2)	0.0330 (11)	
H18	1.309315	0.431529	0.360768	0.040*	
C19	1.1693 (5)	0.3598 (3)	0.3952 (2)	0.0292 (10)	
H19	1.218027	0.315628	0.385936	0.035*	
C1	0.3895 (7)	0.4989 (4)	0.5682 (3)	0.0584 (17)	
H1A	0.340100	0.507364	0.526281	0.088*	
H1B	0.492426	0.497970	0.565103	0.088*	
H1C	0.366186	0.538785	0.596796	0.088*	
O1	0.3467 (5)	0.4319 (3)	0.5916 (2)	0.0596 (12)	
H1	0.414572	0.412812	0.614234	0.089*	

### Bis(triphenylsulfonium) tetrachloridomercury(II) methanol monosolvate (III) Atomic displacement parameters (Å^2^)

**Table:**

	*U*^11^	*U*^22^	*U*^33^	*U*^12^	*U*^13^	*U*^23^
Cl1	0.0466 (6)	0.0291 (6)	0.0259 (5)	0.0051 (5)	0.0120 (5)	0.0060 (4)
Hg1	0.02457 (10)	0.02156 (10)	0.02097 (10)	−0.00267 (6)	0.00126 (7)	0.00035 (6)
Cl2	0.0311 (5)	0.0242 (5)	0.0322 (5)	−0.0075 (4)	0.0000 (4)	−0.0037 (4)
Cl3	0.0245 (5)	0.0308 (5)	0.0282 (5)	−0.0029 (4)	−0.0029 (4)	0.0062 (4)
Cl4	0.0331 (6)	0.0356 (6)	0.0431 (7)	−0.0021 (5)	0.0143 (5)	−0.0141 (5)
S2	0.0189 (4)	0.0194 (5)	0.0218 (5)	−0.0003 (4)	0.0010 (4)	0.0019 (4)
C20	0.0184 (19)	0.023 (2)	0.021 (2)	−0.0013 (16)	−0.0003 (15)	−0.0010 (17)
C21	0.029 (2)	0.022 (2)	0.031 (2)	−0.0004 (18)	0.0063 (18)	0.0032 (18)
C22	0.031 (2)	0.026 (2)	0.047 (3)	0.0064 (19)	0.009 (2)	−0.001 (2)
C23	0.026 (2)	0.033 (3)	0.036 (3)	0.0004 (19)	0.0085 (19)	−0.003 (2)
C24	0.028 (2)	0.029 (2)	0.030 (2)	−0.0072 (19)	0.0030 (18)	0.0010 (19)
C25	0.027 (2)	0.021 (2)	0.023 (2)	−0.0024 (17)	0.0008 (17)	0.0006 (17)
C26	0.021 (2)	0.018 (2)	0.026 (2)	0.0020 (16)	0.0043 (16)	0.0026 (17)
C27	0.029 (2)	0.027 (2)	0.032 (2)	−0.0023 (19)	−0.0005 (19)	−0.002 (2)
C28	0.042 (3)	0.021 (2)	0.042 (3)	−0.001 (2)	0.009 (2)	−0.002 (2)
C29	0.030 (2)	0.026 (2)	0.040 (3)	0.0071 (19)	0.009 (2)	0.008 (2)
C30	0.023 (2)	0.036 (3)	0.039 (3)	0.0007 (19)	0.000 (2)	0.013 (2)
C31	0.022 (2)	0.026 (2)	0.027 (2)	−0.0020 (17)	0.0034 (17)	0.0018 (18)
C32	0.024 (2)	0.026 (2)	0.021 (2)	0.0039 (17)	−0.0018 (17)	−0.0014 (17)
C33	0.028 (2)	0.043 (3)	0.024 (2)	−0.004 (2)	0.0021 (18)	−0.002 (2)
C34	0.035 (3)	0.040 (3)	0.031 (3)	−0.004 (2)	−0.002 (2)	−0.004 (2)
C35	0.043 (3)	0.038 (3)	0.022 (2)	0.008 (2)	−0.003 (2)	−0.006 (2)
C36	0.038 (3)	0.059 (4)	0.028 (3)	0.005 (2)	0.011 (2)	0.004 (2)
C37	0.025 (2)	0.048 (3)	0.029 (2)	−0.001 (2)	0.0040 (19)	0.000 (2)
S1	0.0190 (5)	0.0252 (5)	0.0213 (5)	−0.0003 (4)	0.0013 (4)	−0.0013 (4)
C2	0.0178 (19)	0.025 (2)	0.026 (2)	−0.0011 (16)	0.0018 (16)	0.0002 (18)
C3	0.025 (2)	0.028 (2)	0.024 (2)	0.0000 (18)	0.0002 (17)	−0.0002 (18)
C4	0.021 (2)	0.040 (3)	0.038 (3)	−0.0031 (19)	−0.0011 (19)	0.002 (2)
C5	0.019 (2)	0.043 (3)	0.044 (3)	−0.001 (2)	0.008 (2)	0.003 (2)
C6	0.031 (2)	0.045 (3)	0.029 (2)	0.002 (2)	0.011 (2)	−0.001 (2)
C7	0.023 (2)	0.043 (3)	0.026 (2)	0.002 (2)	−0.0006 (18)	−0.004 (2)
C8	0.0183 (19)	0.026 (2)	0.026 (2)	−0.0001 (17)	0.0038 (16)	0.0009 (18)
C9	0.025 (2)	0.033 (3)	0.035 (3)	−0.0053 (19)	−0.0006 (19)	0.001 (2)
C10	0.039 (3)	0.037 (3)	0.047 (3)	0.000 (2)	0.009 (2)	0.017 (2)
C11	0.031 (2)	0.045 (3)	0.024 (2)	0.009 (2)	0.0032 (19)	0.010 (2)
C12	0.028 (2)	0.041 (3)	0.026 (2)	0.005 (2)	0.0000 (18)	−0.003 (2)
C13	0.027 (2)	0.029 (2)	0.024 (2)	0.0037 (18)	0.0017 (17)	−0.0002 (18)
C14	0.025 (2)	0.026 (2)	0.019 (2)	−0.0016 (17)	−0.0023 (16)	−0.0013 (17)
C15	0.032 (2)	0.028 (2)	0.038 (3)	−0.001 (2)	0.012 (2)	−0.003 (2)
C16	0.047 (3)	0.025 (2)	0.039 (3)	0.002 (2)	0.012 (2)	−0.004 (2)
C17	0.046 (3)	0.032 (3)	0.025 (2)	−0.012 (2)	0.008 (2)	−0.001 (2)
C18	0.027 (2)	0.043 (3)	0.030 (2)	−0.004 (2)	0.0061 (19)	−0.001 (2)
C19	0.026 (2)	0.034 (3)	0.029 (2)	0.0008 (18)	0.0043 (19)	0.0024 (19)
C1	0.042 (3)	0.072 (5)	0.061 (4)	0.004 (3)	0.003 (3)	0.009 (3)
O1	0.048 (2)	0.062 (3)	0.068 (3)	−0.009 (2)	0.002 (2)	−0.012 (2)

### Bis(triphenylsulfonium) tetrachloridomercury(II) methanol monosolvate (III) Geometric parameters (Å, º)

**Table:**

Cl1—Hg1	2.5333 (11)	S1—C8	1.789 (4)
Hg1—Cl2	2.4602 (10)	S1—C14	1.796 (4)
Hg1—Cl3	2.4773 (10)	C2—C3	1.388 (6)
Hg1—Cl4	2.4657 (11)	C2—C7	1.397 (6)
S2—C20	1.783 (4)	C3—H3	0.9500
S2—C26	1.783 (4)	C3—C4	1.382 (6)
S2—C32	1.784 (4)	C4—H4	0.9500
C20—C21	1.395 (6)	C4—C5	1.376 (7)
C20—C25	1.390 (6)	C5—H5	0.9500
C21—H21	0.9500	C5—C6	1.387 (7)
C21—C22	1.389 (6)	C6—H6	0.9500
C22—H22	0.9500	C6—C7	1.382 (7)
C22—C23	1.380 (7)	C7—H7	0.9500
C23—H23	0.9500	C8—C9	1.379 (7)
C23—C24	1.382 (7)	C8—C13	1.380 (6)
C24—H24	0.9500	C9—H9	0.9500
C24—C25	1.385 (6)	C9—C10	1.382 (7)
C25—H25	0.9500	C10—H10	0.9500
C26—C27	1.392 (6)	C10—C11	1.394 (8)
C26—C31	1.386 (6)	C11—H11	0.9500
C27—H27	0.9500	C11—C12	1.384 (7)
C27—C28	1.385 (7)	C12—H12	0.9500
C28—H28	0.9500	C12—C13	1.392 (6)
C28—C29	1.382 (7)	C13—H13	0.9500
C29—H29	0.9500	C14—C15	1.376 (6)
C29—C30	1.377 (7)	C14—C19	1.384 (6)
C30—H30	0.9500	C15—H15	0.9500
C30—C31	1.385 (6)	C15—C16	1.385 (7)
C31—H31	0.9500	C16—H16	0.9500
C32—C33	1.380 (6)	C16—C17	1.386 (7)
C32—C37	1.390 (6)	C17—H17	0.9500
C33—H33	0.9500	C17—C18	1.387 (7)
C33—C34	1.384 (7)	C18—H18	0.9500
C34—H34	0.9500	C18—C19	1.388 (7)
C34—C35	1.375 (7)	C19—H19	0.9500
C35—H35	0.9500	C1—H1A	0.9800
C35—C36	1.383 (8)	C1—H1B	0.9800
C36—H36	0.9500	C1—H1C	0.9800
C36—C37	1.394 (7)	C1—O1	1.382 (8)
C37—H37	0.9500	O1—H1	0.8400
S1—C2	1.790 (4)		
			
Cl2—Hg1—Cl1	104.45 (4)	C8—S1—C2	102.6 (2)
Cl2—Hg1—Cl3	117.50 (4)	C8—S1—C14	105.5 (2)
Cl2—Hg1—Cl4	112.63 (4)	C3—C2—S1	115.8 (3)
Cl3—Hg1—Cl1	110.82 (4)	C3—C2—C7	121.7 (4)
Cl4—Hg1—Cl1	109.71 (4)	C7—C2—S1	122.5 (3)
Cl4—Hg1—Cl3	101.77 (4)	C2—C3—H3	120.7
C20—S2—C26	106.8 (2)	C4—C3—C2	118.6 (4)
C20—S2—C32	104.2 (2)	C4—C3—H3	120.7
C26—S2—C32	102.9 (2)	C3—C4—H4	119.6
C21—C20—S2	114.7 (3)	C5—C4—C3	120.8 (4)
C25—C20—S2	123.2 (3)	C5—C4—H4	119.6
C25—C20—C21	122.1 (4)	C4—C5—H5	120.0
C20—C21—H21	120.7	C4—C5—C6	120.1 (4)
C22—C21—C20	118.7 (4)	C6—C5—H5	120.0
C22—C21—H21	120.7	C5—C6—H6	119.6
C21—C22—H22	120.1	C7—C6—C5	120.7 (5)
C23—C22—C21	119.8 (4)	C7—C6—H6	119.6
C23—C22—H22	120.1	C2—C7—H7	120.9
C22—C23—H23	119.7	C6—C7—C2	118.2 (4)
C22—C23—C24	120.7 (4)	C6—C7—H7	120.9
C24—C23—H23	119.7	C9—C8—S1	114.6 (3)
C23—C24—H24	119.5	C9—C8—C13	122.9 (4)
C23—C24—C25	121.0 (4)	C13—C8—S1	122.5 (3)
C25—C24—H24	119.5	C8—C9—H9	120.7
C20—C25—H25	121.1	C8—C9—C10	118.6 (5)
C24—C25—C20	117.7 (4)	C10—C9—H9	120.7
C24—C25—H25	121.1	C9—C10—H10	120.1
C27—C26—S2	122.7 (3)	C9—C10—C11	119.9 (5)
C31—C26—S2	115.6 (3)	C11—C10—H10	120.1
C31—C26—C27	121.7 (4)	C10—C11—H11	119.8
C26—C27—H27	120.7	C12—C11—C10	120.4 (4)
C28—C27—C26	118.6 (4)	C12—C11—H11	119.8
C28—C27—H27	120.7	C11—C12—H12	119.9
C27—C28—H28	119.9	C11—C12—C13	120.3 (5)
C29—C28—C27	120.2 (4)	C13—C12—H12	119.9
C29—C28—H28	119.9	C8—C13—C12	117.9 (4)
C28—C29—H29	119.7	C8—C13—H13	121.0
C30—C29—C28	120.5 (4)	C12—C13—H13	121.0
C30—C29—H29	119.7	C15—C14—S1	121.8 (4)
C29—C30—H30	119.7	C15—C14—C19	121.9 (4)
C29—C30—C31	120.6 (4)	C19—C14—S1	116.2 (3)
C31—C30—H30	119.7	C14—C15—H15	120.5
C26—C31—H31	120.8	C14—C15—C16	118.9 (4)
C30—C31—C26	118.4 (4)	C16—C15—H15	120.5
C30—C31—H31	120.8	C15—C16—H16	119.8
C33—C32—S2	123.2 (3)	C15—C16—C17	120.5 (5)
C33—C32—C37	121.7 (4)	C17—C16—H16	119.8
C37—C32—S2	115.0 (3)	C16—C17—H17	120.2
C32—C33—H33	120.5	C16—C17—C18	119.6 (5)
C32—C33—C34	119.0 (4)	C18—C17—H17	120.2
C34—C33—H33	120.5	C17—C18—H18	119.7
C33—C34—H34	119.9	C17—C18—C19	120.5 (4)
C35—C34—C33	120.3 (5)	C19—C18—H18	119.7
C35—C34—H34	119.9	C14—C19—C18	118.5 (4)
C34—C35—H35	119.7	C14—C19—H19	120.7
C34—C35—C36	120.6 (4)	C18—C19—H19	120.7
C36—C35—H35	119.7	H1A—C1—H1B	109.5
C35—C36—H36	120.0	H1A—C1—H1C	109.5
C35—C36—C37	120.0 (5)	H1B—C1—H1C	109.5
C37—C36—H36	120.0	O1—C1—H1A	109.5
C32—C37—C36	118.4 (5)	O1—C1—H1B	109.5
C32—C37—H37	120.8	O1—C1—H1C	109.5
C36—C37—H37	120.8	C1—O1—H1	109.5
C2—S1—C14	104.2 (2)		
			
S2—C20—C21—C22	−176.8 (4)	S1—C2—C3—C4	−177.7 (4)
S2—C20—C25—C24	177.0 (3)	S1—C2—C7—C6	178.0 (4)
S2—C26—C27—C28	−177.9 (4)	S1—C8—C9—C10	−175.4 (4)
S2—C26—C31—C30	178.7 (4)	S1—C8—C13—C12	175.8 (3)
S2—C32—C33—C34	177.7 (4)	S1—C14—C15—C16	−177.0 (4)
S2—C32—C37—C36	−177.7 (4)	S1—C14—C19—C18	179.1 (4)
C20—S2—C26—C27	−73.2 (4)	C2—S1—C8—C9	85.4 (4)
C20—S2—C26—C31	109.0 (3)	C2—S1—C8—C13	−92.3 (4)
C20—S2—C32—C33	20.3 (5)	C2—S1—C14—C15	6.7 (4)
C20—S2—C32—C37	−161.1 (4)	C2—S1—C14—C19	−170.1 (3)
C20—C21—C22—C23	−0.3 (7)	C2—C3—C4—C5	−0.8 (7)
C21—C20—C25—C24	−1.1 (6)	C3—C2—C7—C6	−1.1 (7)
C21—C22—C23—C24	−1.3 (8)	C3—C4—C5—C6	−0.4 (8)
C22—C23—C24—C25	1.7 (7)	C4—C5—C6—C7	0.8 (8)
C23—C24—C25—C20	−0.5 (7)	C5—C6—C7—C2	−0.1 (8)
C25—C20—C21—C22	1.5 (7)	C7—C2—C3—C4	1.5 (7)
C26—S2—C20—C21	−158.3 (3)	C8—S1—C2—C3	−156.0 (3)
C26—S2—C20—C25	23.4 (4)	C8—S1—C2—C7	24.9 (4)
C26—S2—C32—C33	−91.1 (4)	C8—S1—C14—C15	−100.9 (4)
C26—S2—C32—C37	87.5 (4)	C8—S1—C14—C19	82.3 (4)
C26—C27—C28—C29	−0.6 (7)	C8—C9—C10—C11	−0.8 (8)
C27—C26—C31—C30	0.8 (7)	C9—C8—C13—C12	−1.7 (7)
C27—C28—C29—C30	0.7 (8)	C9—C10—C11—C12	−1.2 (8)
C28—C29—C30—C31	−0.1 (8)	C10—C11—C12—C13	1.8 (7)
C29—C30—C31—C26	−0.6 (7)	C11—C12—C13—C8	−0.4 (7)
C31—C26—C27—C28	−0.2 (7)	C13—C8—C9—C10	2.3 (7)
C32—S2—C20—C21	93.2 (4)	C14—S1—C2—C3	94.3 (4)
C32—S2—C20—C25	−85.1 (4)	C14—S1—C2—C7	−84.9 (4)
C32—S2—C26—C27	36.2 (4)	C14—S1—C8—C9	−165.8 (3)
C32—S2—C26—C31	−141.6 (3)	C14—S1—C8—C13	16.5 (4)
C32—C33—C34—C35	0.3 (8)	C14—C15—C16—C17	−1.5 (8)
C33—C32—C37—C36	0.9 (8)	C15—C14—C19—C18	2.3 (7)
C33—C34—C35—C36	0.2 (8)	C15—C16—C17—C18	1.4 (8)
C34—C35—C36—C37	−0.1 (8)	C16—C17—C18—C19	0.7 (7)
C35—C36—C37—C32	−0.4 (8)	C17—C18—C19—C14	−2.5 (7)
C37—C32—C33—C34	−0.8 (8)	C19—C14—C15—C16	−0.4 (7)

### Bis(triphenylsulfonium) tetrachloridomercury(II) methanol monosolvate (III) Hydrogen-bond geometry (Å, º)

**Table:**

*D*—H···*A*	*D*—H	H···*A*	*D*···*A*	*D*—H···*A*
O1—H1···Cl1	0.84	2.41	3.187 (5)	155
C3—H3···Cl2^i^	0.95	2.75	3.631 (4)	155
C10—H10···Cl4^ii^	0.95	2.65	3.588 (6)	168
C13—H13···O1^iii^	0.95	2.56	3.192 (7)	124
C19—H19···Cl3^i^	0.95	2.64	3.542 (5)	159
C21—H21···Cl3	0.95	2.72	3.639 (4)	163
C31—H31···Cl1	0.95	2.81	3.630 (5)	145
C36—H36···Cl4^iv^	0.95	2.62	3.573 (5)	178
C37—H37···Cl4	0.95	2.81	3.712 (5)	159

Symmetry codes: (i) *x*+1/2, −*y*+1/2, *z*−1/2; (ii) −*x*+3/2, *y*−1/2, −*z*+3/2; (iii) *x*+1, *y*, *z*; (iv) −*x*+1, −*y*+1, −*z*+2.
